# Entropy predicts sensitivity of pseudorandom seeds

**DOI:** 10.1101/gr.277645.123

**Published:** 2023-07

**Authors:** Benjamin Dominik Maier, Kristoffer Sahlin

**Affiliations:** Department of Mathematics, Stockholm University, 106 91 Stockholm, Sweden

## Abstract

Seed design is important for sequence similarity search applications such as read mapping and average nucleotide identity (ANI) estimation. Although *k*-mers and spaced *k*-mers are likely the most well-known and used seeds, sensitivity suffers at high error rates, particularly when indels are present. Recently, we developed a pseudorandom seeding construct, strobemers, which was empirically shown to have high sensitivity also at high indel rates. However, the study lacked a deeper understanding of why. In this study, we propose a model to estimate the entropy of a seed and find that seeds with high entropy, according to our model, in most cases have high match sensitivity. Our discovered seed randomness–sensitivity relationship explains why some seeds perform better than others, and the relationship provides a framework for designing even more sensitive seeds. We also present three new strobemer seed constructs: mixedstrobes, altstrobes, and multistrobes. We use both simulated and biological data to show that our new seed constructs improve sequence-matching sensitivity to other strobemers. We show that the three new seed constructs are useful for read mapping and ANI estimation. For read mapping, we implement strobemers into minimap2 and observe 30% faster alignment time and 0.2% higher accuracy than using *k*-mers when mapping reads at high error rates. As for ANI estimation, we find that higher entropy seeds have a higher rank correlation between estimated and true ANI.

## Introduction

Short *k*-length substrings of a sequence, often referred to as *k*-mers, are widely used for sequence comparison in bioinformatic applications. A *k*-mer that is shared by two sequences implies an identical region of size *k*, and with appropriate length on *k*, we may detect similar but nonidentical regions through shared *k*-mers. Some of the reasons that *k*-mers are often used for sequence similarity detection is because they are fast to construct and because their fixed length is easy to represent, store, and query, for example, with hash tables or more succinct data structures such as bloom filters (Bloom 1970), the FM-index (Ferragina and Manzini 2000), and many more (Marchet et al. 2021). As *k*-mers indicate shared sequences, they are often used as markers, or *seeds*, indicating regions for more extensive similarity comparison, for example, through pairwise alignment.

With the broad use of *k*-mers as seeds, several limitations have also been identified. For example, *k*-mers are sensitive to mutations. If *k* is too small, we may obtain many redundant hits (e.g., owing to repeats). On the other hand, a too large *k* may destroy all matches (low *sensitivity*) in mutation-dense regions or error-prone reads. Detailed modeling of *k*-mers’ sensitivity to substitutions at different rates was performed by Blanca et al. (2022). Some studies have proposed altering the underlying biological sequence to reduce the mutation rates with, for example, homopolymer compression (Au et al. 2012), or modifying the mutation distribution using more advanced sequence transformations (Blassel et al. 2022). However, most work has been aimed at increasing seed sensitivity and lower seed repetitiveness of *k*-mers by proposing alternative *seed constructs*.

### Other seed constructs

Some approaches aim to alleviate repetitiveness issues in downstream analysis by dynamically extending the *k*-mers to provide a less redundant set of matching seeds such as maximal exact matches (MEMs), maximal unique matches (MUMs) (Delcher et al. 1999), MCAS (Jain et al. 2022), and context-aware seeds (Xin et al. 2020). These seeding constructs have been referred to as dynamic seeds (Sahlin et al. 2023) as they are fixed neither in length nor in the number of CPU cycles for their construction. There are also seeding constructs known as *subsampling methods* that aim to use only a subsample of *k*-mers as seeds owing to their redundant nature using, for example, minimizers (Roberts et al. 2004) or later subsampling techniques (DeBlasio et al. 2019; Ekim et al. 2020; Edgar 2021; Frith et al. 2021; Zheng et al. 2021; Frith Shaw et al. 2023). For an extensive study of subsampling techniques, see the work by Shaw and Yu (2021).

To overcome the issue of requiring only exact matches, spaced seeds (or spaced *k*-mers) (Ma et al. 2002), vector seeds (Brejová et al. 2005), covering template families (Giladi et al. 2010), insertion/deletion (indel) seeds (Mak et al. 2006), and SimHash-based constructs (Charikar 2002) such as permutation-based seeds (Lederman 2013) or BLEND (Firtina et al. 2023) have been proposed that, particularly, tolerate substitutions. Covering template families and indel-seeds are also designed to match over small indels and are based on extracting several fixed-pattern seeds per query position. For example, in the work by Giladi et al. (2010), a combination of patterns is chosen to provide a guarantee that at least one seed matches. The required number of extracted seeds increases with indel size. Seeds that do not require an identical sequence to match are often called *fuzzy seeds*. In applications in which substitutions are frequent, spaced *k*-mers have had practical success and are used in several state-of-the-art applications, such as in the general sequence similarity search software BLAST (Altschul et al. 1990), and for metagenomic classification (Břinda et al. 2015) and long-read mapping (Sović et al. 2016).

### Previous work on seed sensitivity

In spaced seed literature, seed sensitivity has been extensively studied. Typically, when using seeds, an alignment is triggered if a certain number of seeds match in a region, for example, through requiring either multiple *hits* (seed matches) (Burkhardt et al. 1999) or a single hit (Keich et al. 2004) in a region. One drawback of requiring multiple hits is that a threshold does not distinguish highly overlapping hits from disjoint ones. For this reason, seed coverage (union of matching positions in a region) has been proposed (Noé and Martin 2014). As spaced seeds patterns are fixed, using multiple patterns as opposed to a single seed pattern can increase sensitivity at low cost in specificity (Sun and Buhler 2005).

The main conclusion in spaced seed literature is that many highly overlapping matches are redundant, are uninformative, and can lead to unnecessary computations for sequence-matching applications. Typically, the aim is to select a set of seed patterns consisting of fixed and wildcard positions that overlap or correlate as little as possible. Related work on minimizing the overlap of hits has been studied in the form of clump statistics (Stefanov et al. 2007) or *overlap complexity* (Ilie and Ilie 2007), the average distance between successive nonoverlapping hits (Yang and Zhang 2008). In addition, there are other theoretical studies of seed sensitivity quantifying the correlation between seeds (Kong 2007) or using generating functions from analytical combinatorics (Filion 2018), which have also been used in practice to select suitably spaced seeds when mapping short reads (Filion et al. 2020). Finally, if seed sensitivity is defined as the fraction of hits across a target sequence, merely computing the sensitivity of a spaced seed pattern analytically is challenging (Kucherov et al. 2006).

### Strobemers and pseudorandomness

**Figure 1.**
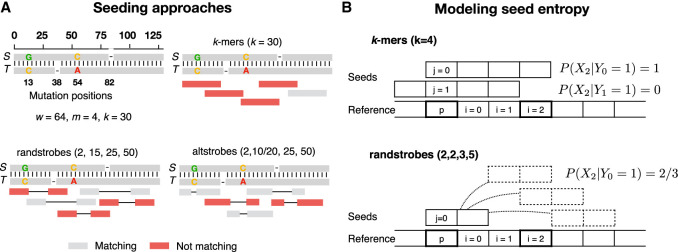
Sampling of *k*-mers, randstrobes, and altstrobes. Panel *A* shows two homologous sequences *S* and *T* containing four mutations with *k*-mers, randstrobes, and altstrobes sampled from *T*. When the sampled positions in a seed appear more random, it is likelier that at least one of the seeds in the region will sample only non-mutated positions with respect to *S*. Such non-mutated seeds are colored gray in the figure, and seeds containing mutated positions with respect to *S* are shown in red. Sampling non-mutated seeds in necessary to produce a seed match with seeds from *S*. Only a subset of five seeds in the region is shown for clarity. In the example, the seed constructs all sample 30 fixed positions in a window of *w* = 64 nucleotides which is the maximal span of a randstrobe with parameters (2,15,25,50). The mutations are distributed such that they destroy all shared *k*-mers in the region, and most of the randstrobes. Altstrobes have the possibility to sample *k*-mers of two different lengths at each site, which allow them a higher probability to match between mutations. Panel *B* illustrates our modeling of seed entropy for seed construct *k*-mers and randstrobes. In the case of *k*-mers, there is no pseudorandomness and therefore all probabilities are either 0 or 1, leading to an entropy of 0. Under uniform hashing, the randstrobes will have a probability of 2/3 of sampling position *i* owing to the three possible sampling positions of the second strobe. Boldfaced squares indicate the positions considered for the computation of probabilities shown in the figure.

Recently, we introduced a new class of fuzzy seed constructs, *strobemers* (Sahlin 2021), tolerant to substitutions and indels. Strobemers expand on the ideas of neighboring minimizer pairs (Chin and Khalak 2019; Sahlin and Medvedev 2021) and *k*-min-mers (Ekim et al. 2021). Strobemers are constructed by linking together a set of smaller *k*-mers, called *strobes*, with different methods to link the strobes (minstrobes, randstrobes, hybridstrobes). The link methods use pseudorandom hash functions to decide the strobes to sample in a seed. Therefore, strobemers are what we in this study call *pseudorandom* seeds. We use the term pseudorandom to refer to the characteristic that the sampling profile of the strobes may appear random to the eye but is deterministic given the hash function, similar to random number generators. Deterministic sampling is necessary to generate matches between homologous sequences. Figure 1A shows randstrobe seeds where the distance between the strobes appears random but is deterministic given the hash function and the underlying sequence. For how the pseudorandom sampling works, see the Methods section. We use the term *pseudorandom* seeds to distinguish them from other types of fuzzy seeds that do not have a pseudorandom sampling process, for example, SimHash based on locality sensitive hashing techniques (Charikar 2002). It was shown that strobemers could offer higher sensitivity and lower repetitiveness over *k*-mers, and they have been used for short-read mapping (Sahlin 2022), long-read overlap detection (Firtina et al. 2023), transcriptomic long-read normalization (Nip et al. 2023), and symbiont classification with long reads (Xu et al. 2023).

### Motivation and aim

Without a clear metric to optimize, seed design ultimately involves trial-and-error-based analysis by plugging different seeds in alignment algorithms and evaluating the alignment results. Such analysis takes time and requires substantial computation resources. More critically, such trial-and-error-based experiments may not provide fair feedback. Aligners consist of downstream heuristics to evaluate candidate mapping sites and have seed-specific parameters carefully tailed for the seed they were initially designed for. Therefore, a new seed could reach higher accuracy or faster alignment time if its implementation was centered around another seeding construct. This makes trial-and-error-based analysis inhibit the development of better seeding techniques and motivates the study of performance metrics more directly connected to the seed itself.

Although the spaced seed literature has identified metrics that predict high sensitivity for spaced seeds, it has so far not been studied for pseudorandom seeds such as strobemers. The aforementioned spaced-seed studies are all based on seeds with a fixed sampling pattern (e.g., *k*-mers and spaced *k*-mers) in which the sampling decision has no pseudorandom behavior after the seed pattern has been chosen. Also, although there are spaced seed studies focusing on optimal seed selection of a single seed pattern (Gotea et al. 2003), most aforementioned spaced seed studies are centered around selecting a set of spaced seeds with complementary properties.

Inspired by spaced *k*-mer literature that has focused on understanding the mechanics of high sensitivity seeds, we aimed to find why pseudorandom seeds such as strobemers achieve high sensitivity and whether we could find a metric to predict it. In work by Sahlin (2021), it was shown that randstrobes and hybridstrobes had higher sensitivity than *k*-mers, spaced *k*-mers, and minstrobes. These two constructs have, unlike the rest, a pseudorandom component in how they select the next strobe, creating a seemingly more random seed coverage distribution (for an example of randstrobes and *k*-mers, see Fig. 1A). Therefore, it stands to reason that something in the randomness of the sampled positions of a seed may be positively correlated with seed sensitivity. We set out to investigate if this was the case.

## Results

### Main contributions and results overview

We modeled the probability that a pseudorandom seed samples a position, given that the seed samples *c* positions in a window of size *w*, called a (*c, w*)-seed (Methods). We further stated the entropy formula for the model (Methods). We also designed three new pseudorandom seed constructs, mixedstrobes, altstrobes, and multistrobes (Methods) and computed the entropy for the new seed constructs as well as the previously proposed seed construct randstrobes. Our main finding is that, among various (*c, w*)-seeds with set parameters on *c* and *w*, the entropy correlates closely with seed sensitivity in most scenarios. We verify that our entropy and sensitivity analysis also selects seeds that produce favorable results in sequence similarity search scenarios using metrics in work by Sahlin (2021). Further, we show that our proposed seed constructs are fast to construct and implement them in minimap2. Even though minimap2 is designed for minimizers and performs subsampling of seeds that distorts the entropy and, thus, our sensitivity predictions, we observe faster alignment time (up to 30%) and slightly higher sensitivity (0.2%) than using *k*-mers of the same size as seeds. We also perform an average nucleotide identity (ANI)–estimation analysis using our strobemer constructs and compare it to estimations from *k*-mers and show that strobemers achieve higher correlation in ANI estimations than *k*-mers and that the highest entropy seeds achieve the best ANI estimations in terms of ranking sequence similarity. We conclude that entropy is a useful metric to optimize (maximize) when constructing pseudorandom seeds, and it avoids trial-and-error-based computational evaluations based on plugging in seed constructs in existing aligners.

### Empirically verifying correlation between seed entropy and sensitivity

We computed the seed entropy for *k*-mers, randstrobes, altstrobes, mixedstrobes, and multistrobes for different parameter settings varying (*k*_*s*_, *k*_*l*_), *q*. Specifically, we used the same parameters as in work by Sahlin (2021), namely, *k* = 30 for *k*-mers and (2, 15, *w*_min_, 50), *w*_min_ ∈ [16, 25, 35, 45] for randstrobes, which gives valid (30, 64)-seeds. We consequently set for mixedstrobes (2, 15, *w*_min_, 50, *q*) with *q* = 0, 0.1, …, 1.0, for altstrobes and multistrobes (2, *k*_*s*_, *k*_*l*_, *w*_min_, 50) with *k*_*s*_ = 1, 2, …, 14. We then evaluated the computed entropy of all seed parametrizations to empirical estimations of seed sensitivity summed over various mutation rates (for simulation details, see Supplemental Sec. S3). The sensitivity is defined as the seed producing at least one match in a contiguous stretch of *w* samples seeds (details in Methods, subsection “Objectives for sequence similarity detection”).

**Figure 2.**
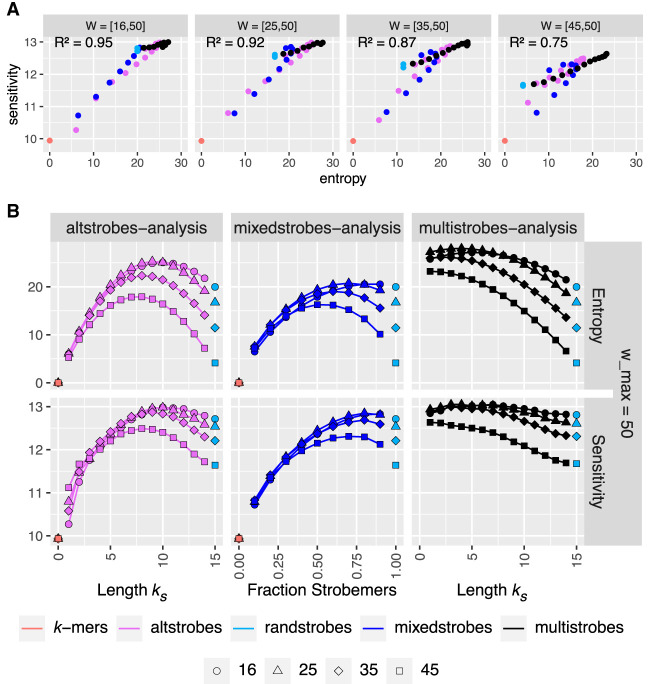
Entropy and Sensitivity relationship of seeds. Panel *A* shows the relationship between entropy (*x*-axis) and sum of sensitivity over different mutation rates (*y*-axis) for various window sizes indicated by the coefficient of determination (square of the Pearson correlation coefficient). Panel *B* shows *H*(*X*|*Y*) (*upper* row) and summed sensitivity over various mutation rates (*lower* row) for *k*-mers, randstrobes, altstrobes for different strobe length parametrizations (*x*-axis) and window sizes (tick mark shapes).

Figure 2A shows the relationship between computed entropy and sensitivity across mutation rates. We observe a very strong correlation (0.96 and 0.92) for seeds computed from window sizes 35 and 26 (i.e., with *w*_min_ of 16 and 25). The correlation weakens as windows get narrower, with only a 0.74 correlation coefficient for very narrow strobe selection window of size 6 (*w*_min_ of 45).

We also wanted to explore which parameter combinations for each seed construct produced the most desired results. Figure 2B shows entropy estimates (top row) compared with the sensitivity (bottom row). Overall, we see that our entropy metric predicts the sensitivity well both between parameters within a seed construct and between seed constructs. Specifically, Figure 2, A and B, shows that our entropy metric captures four trends. First, it suggests that in most cases, narrower (*w*_min_, *w*_max_) leads to lower entropy and, hence, lower sensitivity. This result may seem obvious in hindsight, but it was unknown to us at the time of the strobemers study (Sahlin 2021).

Second, given a fixed *w*_min_, the model typically predicts which parameter settings of *q*, *k*_*s*_ yield good seed sensitivity for the constructs individually. The exception are mixedstrobe entropy peaks for *w*_min_ = 25, 35, and 45, which are slightly shifted to predict peak sensitivity at a lower *q* (0.5–0.9) than what is observed (0.7–0.9). This misprediction is more prevalent with smaller windows such as *w*_min_ = 45 and is also the main contributor for weakening the correlation in Figure 2A (blue dots). We are unable to explain this particular disagreement for mixedstrobes.

Third, we can compare *k*-mers, randstrobes, mixedstrobes, altstrobes, and multistrobes to each other. For most *w*_min_, we observe that multistrobes reach the highest peak entropy, followed in order by altstrobes, mixedstrobes, randstrobes, and, finally, *k*-mers. This trend is also present in the sensitivity curves. The peak sensitivity across all methods (13.0495) was reached by multistrobes with *k*_*s*_ = 4 closely followed by *k*_*s*_ = 3 (13.0494) for *w*_min_ = 25. After that were multistrobes with *k*_*s*_ = 7 for *w*_min_ = 16 (13.048). Several other *k*_*s*_ on the multistrobes curves for *w*_min_ = 16 and 25 also reached a summed sensitivity above 13. For altstrobes, the peak sensitivity (12.980) was reached by *k*_*s*_ = 10 for both *w*_min_ = 16 and *w*_min_ = 25. For mixedstrobes, the peak sensitivity (12.848) was reached by *q* = 0.8 and *w*_min_ = 25. These values roughly agree with the peak entropies of the individual curves. Peak entropy is obtained by multistrobes with *k*_*s*_ = 4 (27.98), agreeing with peak sensitivity.

Fourth, the relative increase in entropy correlates well with the relative increase in sensitivity (Fig. 2A; Supplemental Fig. S1). For example, compare the relative distances between entropy and sensitivity peaks for *k*-mers, randstrobes, altstrobes, mixedstrobes, and multistrobes. Although the entropy curves are, in general, more spread out, the relative distances are relatively well preserved.

Supplemental Figures S1 and S2 show that our observations generally hold also for experiments with *w*_max_ = 100 and 200 with varying *w*_min_. For example, seeds constructed with larger window sizes have a higher correlation between the entropy metric and seed sensitivity (Supplemental Fig. S1). Also, smaller window sizes typically have lower entropy and sensitivity (Supplemental Fig. S2), and the parametrizations for a given seed construct and *w*_min_ can be ranked similarly as for the *w*_max_ = 50 experiments. However, we see a trend that with larger windows, both the sensitivity and entropy curves flatten out, suggesting that variable strobe sizes do not matter as much for sensitivity as for smaller windows. We believe this is because, in a large enough window, there are more possibilities to find error-free stretches longer than the strobe length, diminishing the necessity of hashing short strobes that can fit between mutations.

**Figure 3.**
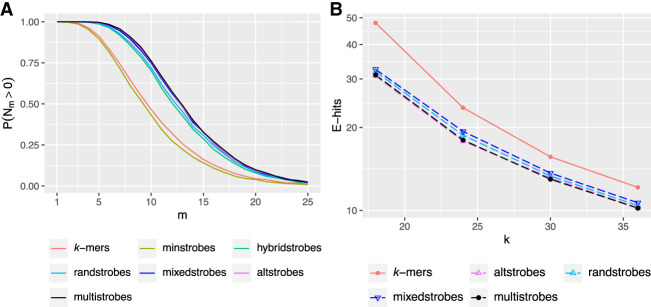
Simulations showing how pseudorandomness in seed construct influences probability of *w* consecutive seeds producing at least one match in a region of length 2*w* = 128 between sequences. Panel *A* shows *P*(*N*_*m*_(30, 64) > 0) for seed constructs *k*-mers (*k* = 30), minstrobes, hybridstrobes, and randstrobes with (2,15,25,50), mixedstrobes (2,15,25,50,0.8), altstrobes (2,10, 20,25,50), and multistrobes (2,5,25,25,50). Each *P*(*N*_*m*_(30, 64) > 0) estimate is derived from 10,000 instances of pairs of strings *S* and *T*. In general, a large gap is observed between non-random constructs (*k*-mers, minstrobes) to constructs with pseudorandomness (hybridstrobes, randstrobes, mixedstrobes, altstrobes) for most mutation frequencies. The total sum of probabilities across *m* is higher for constructs with more random appearance. Panel *B* shows the seed uniqueness as expected number of hits (E-hits) from a seed randomly drawn from human Chromosome 21. Chromosome 21 of the human GRCh38 assembly was seeded with *k*-mers, randstrobes (2, *k*/2, 25, 50), mixedstrobes (2, *k*/2, 25, 50, 0.8), altstrobes (2, *k*/3, 2*k*/3, 25, 50), and multistrobes (2, 5, *k* − 5, 25, 50), whereby the number of extracted nucleotides (*k* = 30) was the same for all seeding techniques.

Lastly, we used the best parametrizations (derived from Fig. 2B) and plotted the sensitivity that the seed constructs achieved when inserting a set of *m* mutations in a fixed length sequence (i.e., giving rise to different mutation rates). The results are shown in Figure 3A. We observe that the sensitivity increase of, for example, multistrobes to randstrobes and *k*-mers is relatively uniformly distributed across the different mutation frequencies.

### Seed uniqueness

Our entropy metric predicts only the sensitivity of seeds. In sequence matching, we also want to control the number of false matches, which is a side effect of fuzzy seeding constructs that have a lower resolution representation of the sequence (i.e., different sequences can produce the same seed). We plotted the *E*-hits metric (expected number of seed hits) for the seed constructs using the best parametrizations of each seeding construct with *w*_min_ = 25 (derived from Fig. 2B). The *E*-hits metric is a measure of how repetitive the seeds are, where lower *E*-hits is better (for details, see Methods) We observe that the increase in sensitivity comes at no cost in uniqueness (Fig. 3B).

### Model limitations

During our study, we learned that entropy is not the only feature that predicts seed sensitivity. An aspect not captured by our model is the probability that a contiguous segment (e.g., strobe or *k*-mer) is destroyed by mutations. An example of why modeling of this is needed is the following. Consider the following two different approaches of sampling mixedstrobes. In approach 1, we sample a *k*-mer or a strobemer based on the hash value of the first *k* nucleotides at the start of the seed. In approach 2, we sample a *k*-mer or a strobemer based on the hash value of the first *k*/2 nucleotides (the strobe length) at the start of the seed. It is straightforward to see that for any mutations *m* > 1, there will be more shared *k/2*-mers than *k*-mers between the sequences. Hence, the probability of sampling the same seed, and therefore of generating a match, is higher for approach 2 (which we implement). The same argument holds for altstrobes and multistrobes, which is why we decide the strobe length based on the hash of *k*_*s*_.

Our model is agnostic to this probability. This becomes apparent when applying our model to strobemers with very narrow window sizes. We computed entropies and sensitivity estimates for mixedstrobes, altstrobes, and multistrobes with window sizes (*w*_min_, *w*_max_) of (49,50), (99,100), and (199,200) (Supplemental Fig. S3). In these cases, the seeds roughly act as spaced *k*-mers but with randomness over strobe size (altstrobes and multistrobes) or strobe fraction (mixedstrobes). Whereas the entropy curves vary and predict clear optima for these window sizes, the sensitivity curves are relatively flat but with peaks for *k*-mers, or near *k*-mer constructs (*k*_*s*_ = 1 altstrobes and mixedstrobes), which is expected when comparing *k*-mers to spaced *k*-mers when indels occur. Any indel within the seeds will destroy the seeds and, thus, give low sensitivity when indels are present at significant fractions. However, our model still estimates positive and variable entropy because of the pseudorandom selection of strobe lengths (for altstrobes and multistrobes) or seed type (for mixedstrobes). The model could therefore be improved by adding a probability distribution over indels or by estimating a probability that a contiguous region is error free.

Also, our model assumes that the function for selecting the position of the downstream strobe has a uniform distribution over the possible positions, which requires a perfectly uniform hash function. This is not true in practice, for example, as we show in Supplemental Figure S4. Also, implementation-specific limitations to achieve uniform hashing for randstrobes have been identified (https://github.com/ksahlin/strobemers/issues/8). Finally, as mentioned, our entropy measure cannot estimate entropy for pseudorandom seed constructs that are correlated between neighboring seeds such as minstrobes and hybridstrobes. However, our results show that the entropy of independent pseudorandom seed constructs as computed by our model overall predicts well the relative sensitivities of pseudorandom seed constructs.

### Sequence-matching results

Furthermore, we used the sequence match analysis performed by Sahlin (2021) to evaluate if the best parametrizations from our entropy–sensitivity analysis also performs well in sequence similarity search scenarios. In the sequence match analysis by Sahlin (2021), several different aspects of sequence-matching performance were evaluated, using both the simulations and genomic Oxford Nanopore Technology (ONT) reads. For details about the data and simulation setup, see Supplemental Section S4. We evaluated altstrobes (*k*_*s*_ = 10), multistrobes (*k*_*s*_ = 5), and mixedstrobes (*q* = 0.1, …, 1.0) against *k*-mers, and the other strobemers. We used *k*_*s*_ = 10 and *k*_*s*_ = 5 for altstrobes and multistrobes, respectively, as according to our entropy and sensitivity analysis, they should be better performing than randstrobes for (*w*_min_, *w*_max_) = (25, 50). First, to verify our results from the sensitivity analysis, we performed an in-depth analysis of altstrobes for different *k*_*s*_ using the match analysis on simulated data performed by Sahlin (2021) (see Supplemental Fig. S5). This analysis confirmed that *k*_*s*_ = 10 was preferable, in concordance with our sensitivity analysis. We then ran the matching analysis on all strobemer constructs. For comparability with the strobemer study (Sahlin 2021), we used the same parameters (e.g., *k* = 30, and strobemers with (2, 15, 25, 50)). We included in the results minstrobes, hybridstrobes, and altstrobes seeded and mixed with *k*-mers at different fractions *q* = 0, 0.1, …, 1.0. We also computed altstrobes with various *k*-mer fractions (i.e., mixed altstrobes) for formatting consistency with the other results. Multistrobes were not seeded and mixed with *k*-mers and therefore always appear at only the fraction *q* = 1.0. We included results for strobemers of orders *n* = 2, 3 and 4, but we focus on evaluating *n* = 2 here.

**Figure 4.**
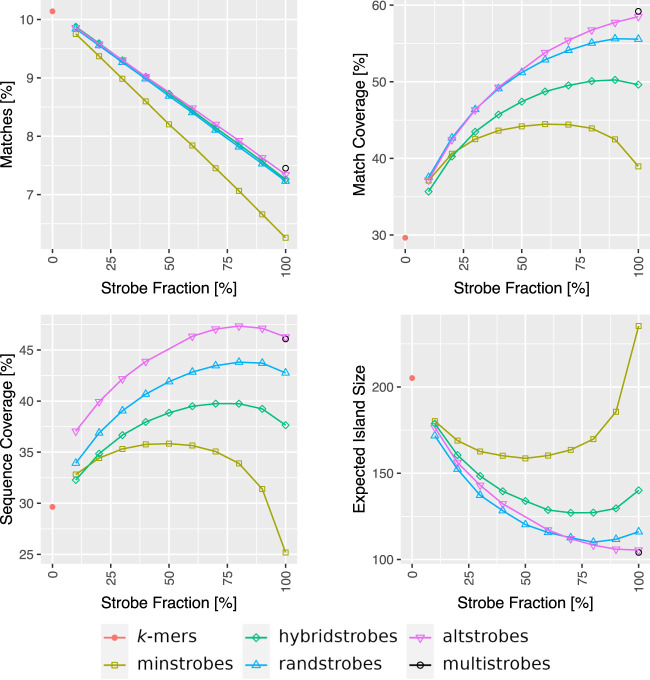
Comparison between (mixed-)strobemers (2,15,25,50, *q*), (mixed-)altstrobes (2,10,20,25,50, *q*), multistrobes (2,5,25,25,50), and *k*-mers (*k* = 30) when mapping genomic Oxford Nanopore Technology (ONT) reads from *E. coli* to its reference. The *E. coli* reads were split up in long disjoint segments of 2000 nt. Next, the segments were seeded with strobemer fractions *q* from 0% (*k*-mers) to 100% (strobemers), downstream windows set to [25,50] and all strobes combined adding up to equal length subsequences of size 30 for better comparison. Then for each segment, the collinear solution of raw hits was computed to subsequently quantify the number of matches, match coverage, sequence coverage, and expected island size.

Both the simulated data (Supplemental Fig. S6, panels with two strobes) and biological data (Fig. 4; Supplemental Figs. S7, S8) experiments confirmed our sensitivity analysis. First, mixedstrobes with a strobemer fraction of ∼70%–80% perform better than strobemer-only seeding, as well in this sequence-matching analysis. The fraction of matches is higher for mixedstrobes at 80% than when seeding only randstrobes, whereas at the same time, the sequence coverage and expected island size were also better. Similar results can be observed for hybridstrobes and minstrobes. Second, altstrobes and multistrobes are outperforming randstrobes and mixedstrobes, whereby multistrobes have the most desired performance on both simulated and biological data, agreeing with our sensitivity analysis. For the biological data, we observe that adding 20% *k*-mers to altstrobes further increases the sequence coverage over only using altstrobes. We believe this is because biological errors are less uniform, which may be beneficial for *k*-mers. As such mixed altstrobes are using 10, 20, and 30 as sampled strobe lengths, it could indicate that using a nonuniform distribution of strobe lengths in multistrobes (as discussed in the sensitivity analysis) could be beneficial.

### Variable substitution frequency models

**Figure 5.**
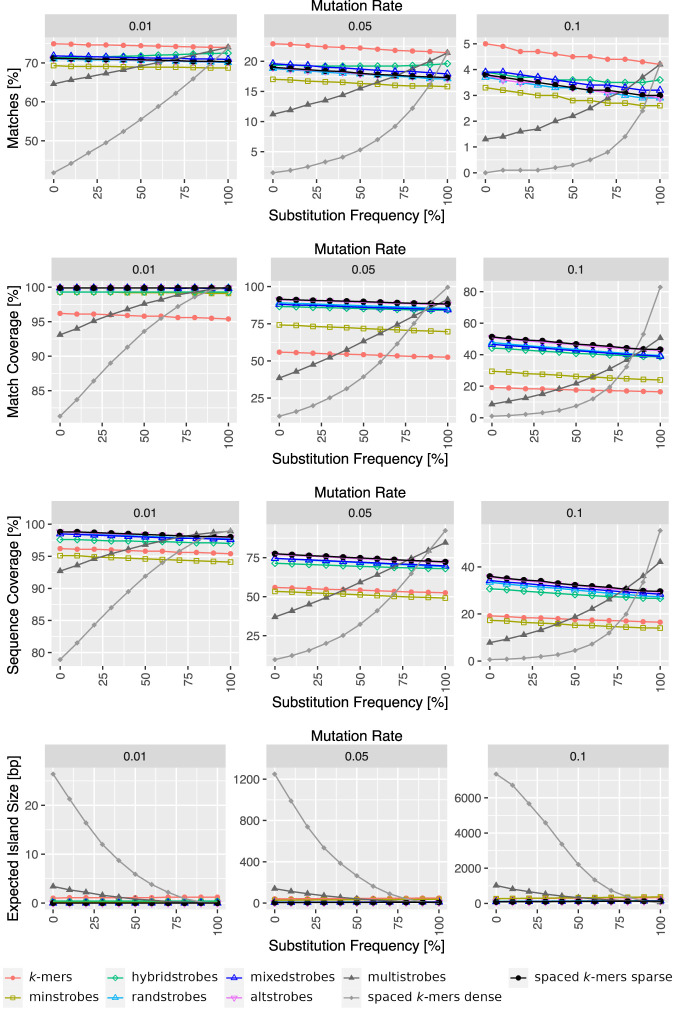
Performance of (spaced) *k*-mers and strobemers with various indel/substitution patterns in simulated data. For all the experiments, 1000 random sequences of length 10,000 nt were created and 1%, 5%, and 10% of the nucleotides of the reference string were mutated for the different experimental conditions. Substitutions were hereby added with probabilities from 0% to 100% (substitution frequency), whereas the other mutation positions were filled with insertions and deletions with equal probability. Subsequently, the sequences were seeded with (spaced) *k*-mers of length *k* = 30 as well as strobemers of order *n* = 2 with all sub-strobes summing up to *k* = 30 and downstream windows set to [25,50].

It is well documented that the frequency of nucleotide substitutions and indels is species specific and can vary across functional components of genomes. Hence, it is important to take these patterns and frequencies into account when benchmarking different seeding approaches. Previous study of strobemers (Sahlin 2021) only investigated equally distributed substitutions, insertions, and deletions at probability 1/3. Here we include an analysis over various substitutions rates (from 0% to 100%), including also spaced *k*-mer seeds constructed as in work by Sahlin (2021). Our analysis show that although spaced *k*-mers are the method of choice when analyzing sequences with low relative fractions of indels (0%–10%), their performance deteriorates as indels become more prevalent (>10%) (Fig. 5). In contrast, the performance of strobemers and *k*-mers are more stable with varying indel rates.

### Strobemers improve ANI estimation

ANI is a metric to estimate pairwise similarity between sequences, commonly used in microbiology to determine whether genomes, contigs, or reads belong to the same species (Ondov et al. 2016; Jain et al. 2018). Although traditional implementations such as that of Goris et al. (2007) required an alignment step that was usually performed using BLAST (Altschul et al. 1990), there are some more recent alignment-free approaches that speed up the estimation greatly without losing significant accuracy (Yoon et al. 2017; Jain et al. 2018). A popular alignment-free metric is the Mash distance (Methods), as used, for example, in Mash (Ondov et al. 2016) and FastANI (Jain et al. 2018).

**Figure 6.**
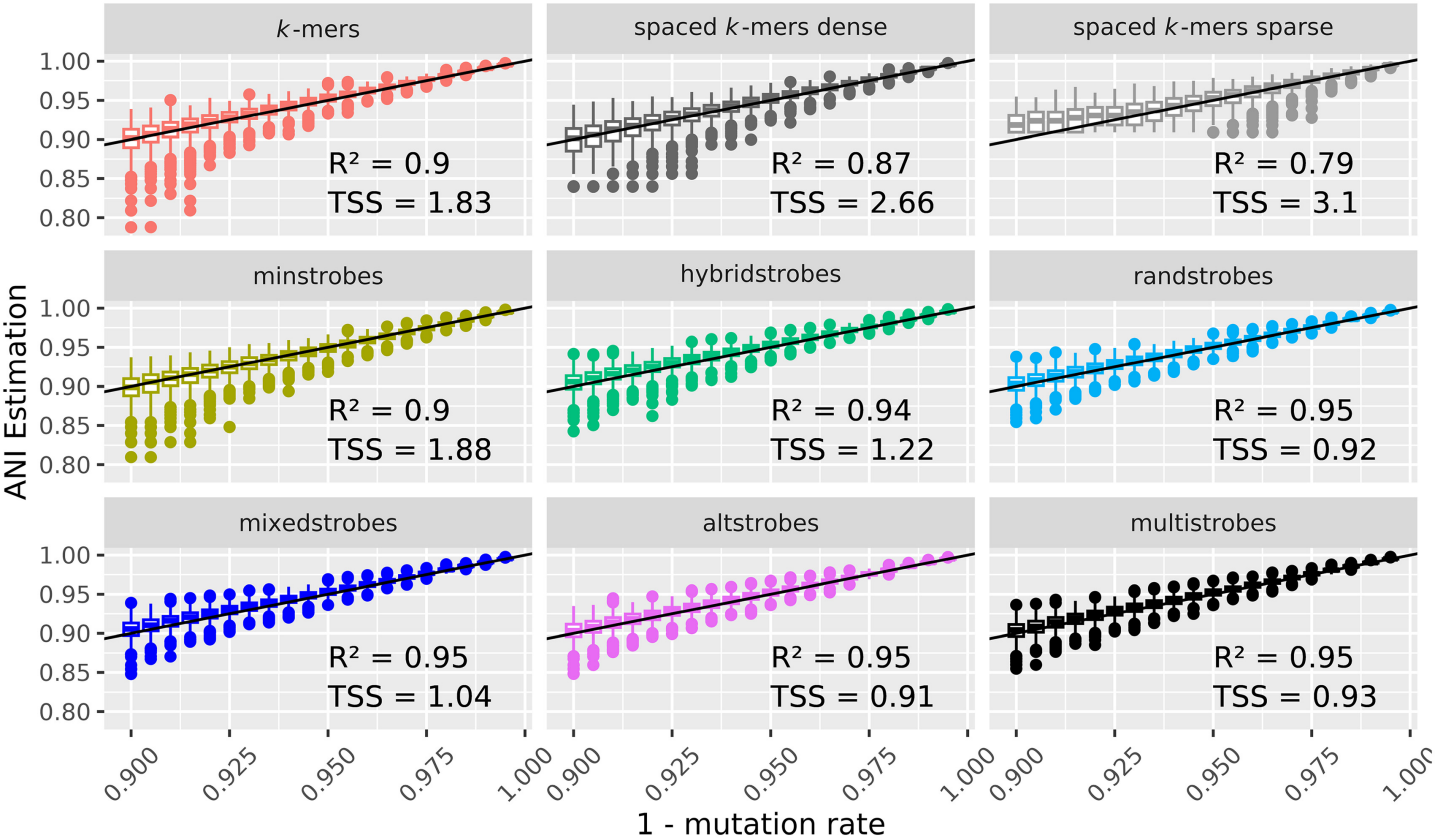
ANI-estimates of simulated sequences. ANI estimations on simulated data as estimated by Adjusted Mash distance (Methods) with *k*-mers, spaced *k*-mers, minstrobes, hybridstrobes, randstrobes (2,15,25,50), mixedstrobes (2,15,25,50,0.8), altstrobes (2,10,20,25,50), and multistrobes (2,5,25,25,50). For each method, the square of the Pearson correlation coefficient (*R*^2^, higher is better) and the total sum of squares (*TSS*; lower is better) between ANI estimation and true mutation rate is given.

**Figure 7.**
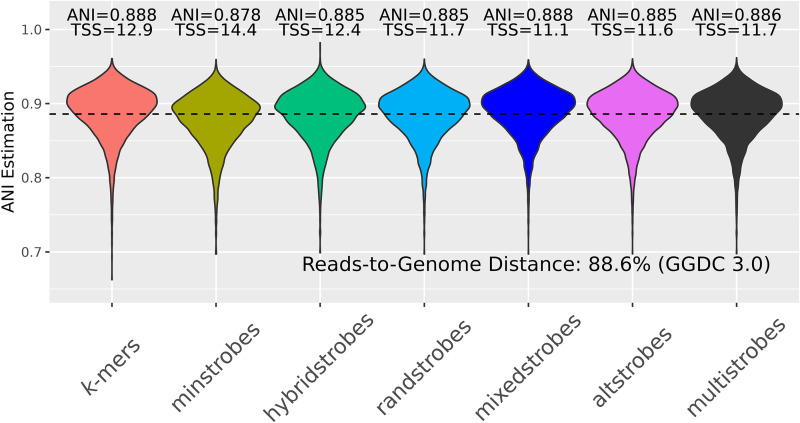
ANI estimation as estimated by Adjusted Mash distance (Methods) between *E. coli* ONT reads and a *E. coli* genome (assembly GCA 003018575.1 ASM301857v1) with *k*-mers, spaced *k*-mers, minstrobes, hybridstrobes, randstrobes (2,15,25,50), mixedstrobes (2,15,25,50,0.8), altstrobes (2,10,20,25,50), and multistrobes (2,5,25,25,50). For each method, the square of the Pearson correlation coefficient (*R*^2^, lower is better) and the TSS (lower is better) between ANI estimation and true mutation rate is given. The ANI was compared to the reads-to-genome distance (88.6%) obtained from GGDC 3.0 with default settings (Meier-Kolthoff et al. 2013, 2022) using the TSS.

We estimated ANI with *k*-mers and our new strobemer constructs with the Mash distance using both simulated data and our ONT reads to benchmark the accuracy of ANI estimations (Methods). We used the *R*^2^ metric (the square of the Pearson correlation coefficient) and the total sum of squares (TSS) to evaluate the approaches. The *R*^2^ metric measures the ranking correlation between the ANI measure and the actual error rate, that is, whether a sequence *A* with lower ANI *B* also gets a lower ANI estimate. This measure is agnostic to how close the ANI estimate is to the true ANI. On the other hand, the TSS measures the actual closeness of the predicted ANI to the true ANI. We observed better *R*^2^ with the randstrobes, mixedstrobes, altstrobes, and multistrobes, indicating that higher entropy seeds are also better at estimating the relative rank of sequences’ various error rates for both the simulated and ONT data (Figs. 6, 7, *R*^2^). As for estimating the actual ANI, the TSS indicated that *k*-mers performed the best. This is not surprising as the Mash distance was designed for *k*-mers and uses *k* in its formula (Methods). However, we modified the Mash distance formula by applying a correction term and fitted the best correction term individually for *k*-mers, spaced *k*-mers, and strobemers (Methods). With the modified Mash distance, TSS indicated that strobemer seeds (particularly randstrobes, altstrobes, and multistrobes) had a better linear correlation between the estimated and true ANI than *k*-mers for both simulated and ONT reads (Figs. 6, 7), albeit the improvement of ANI estimation on the ONT reads is only marginally better. For the ONT data, the true ANI was estimated by the read-to-genome distance obtained from GGDC 3.0 with default settings (Meier-Kolthoff et al. 2013, 2022). Note also that the rank correlation metric *R*^2^ is not affected by adjusting the Mash distance. The rank correlation is better for strobemers under both formulations. In summary, we believe that strobemers seem promising for ANI estimation, particularly for ranking sequence similarity.

### Time and memory to construct altstrobes, mixedstrobes, and multistrobes

**Figure 8.**
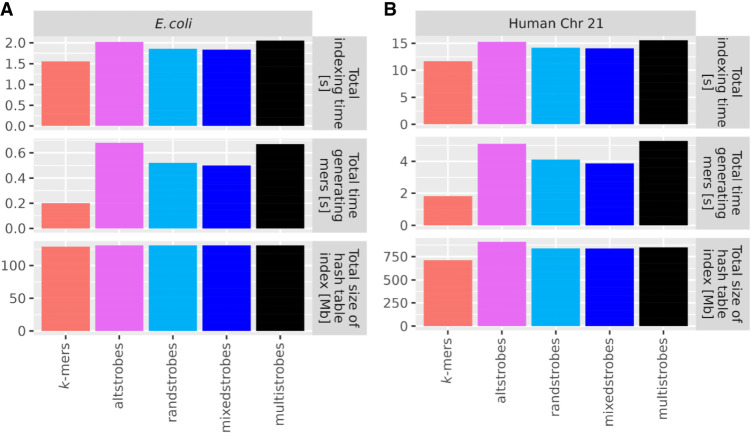
StrobeMap benchmarking. To benchmark our two new seeding techniques, Chromosome 21 of the GRCh38 human genome assembly (*B*) and one *E. coli* genome (assembly GCA 003018575.1 ASM301857v1) (*A*) were indexed with *k*-mers (*k* = 30), randstrobes (2, 15, 25, 50), mixedstrobes (2, 15, 25, 50, 0.8), altstrobes (2, 10, 20, 25, 50), and multistrobes (2, 5, 25, 25, 50) using StrobeMap. All experiments were repeated 10 times and the average (mean) was computed to account for variance in computer processing speed.

We implemented altstrobes, mixedstrobes, and multistrobes in StrobeMap (Sahlin 2021) in C++. *K*-mers are 3.5 times, 3.5 times, and 2.5 times faster than altstrobes, multistrobes, and mixedstrobes, respectively (Fig. 8A,B). However, the time difference becomes negligible when looking at the total indexing time (including, e.g., sorting seeds and adding to hash table), especially when also taking into account that indexing is not the time-limiting factor in most applications. The size on the index is nearly identical (Fig. 8A,B).

### Strobemers in minimap2

We implemented subsampled randstrobes, mixedstrobes, altstrobes, and multistrobes in minimap2 (Li 2018) (see Supplemental Sec. S5) to benchmark the speed and accuracy of our seeding techniques and aligned simulated reads at various error rates to CHM13 (for details, see Supplemental Sec. S4). Our results indicate that altstrobes (2,9,18,25,50), mixedstrobes (2,14,25,50,0.8), and multistrobes (2,6,22,25,50) have slightly more (0.2%) correctly mapped reads and slightly faster alignment time (up to 30%) compared with *k*-mers (*k* = 28) with a similar number of extracted seeds and peak RAM usage (Supplemental Fig. S9). Altstrobes, mixedstrobes, and multistrobes also speed up alignment up to 3.5 times compared with the default setting (*k* = 15). However, the number of correctly mapped reads remains lower than the default setting. This is expected as using much smaller seeds is beneficial for sensitivity at the cost of computing time. Also, minimap2's search and extend parameters are optimized for exact *k*-mer seeds, which may give an implementation-specific advantage to *k*-mers in the analysis. Second, minimap2 uses *k*-mer subsampling through minimizers. Subsampling (also called thinning) is not something we modeled in our analysis and distorts our entropy model, which assumes seeds are sampled from every position.

## Discussion

To our knowledge, we believe that we have provided the first analysis of seed sensitivity for seeds that uses a pseudorandom sampling decision such as strobemers. We describe a model for estimating the entropy (randomness) of a seed, and we discovered a strong positive correlation between the pseudorandomness of a seed construct and its effect on seed sensitivity (Fig. 2A; Supplemental Fig. S2). Our discovered entropy–sensitivity correlation may aid researchers in finding more efficient seed designs. In particular, without a clear metric to optimize, designing efficient seeds may involve trial and error by plugging in a new seed construct in an aligner such as minimap2 and indirectly evaluating the seed performance using the output alignment results as a proxy. First off, this is costly in both time and computations. More pressing, trial-and-error-based experiments may be biased. This is because aligners have many parameters designed and optimized for the seed construct they were developed with, which may obstruct the potential performance gain or even prevent the implementation of a new seed. Our entropy–sensitivity relationship provides a more direct way to predict seed performance and can save time and cost in conducting trial-and-error-based experiments. In some cases, we observed that our metric did not give accurate feedback on sensitivity. We discussed these cases (for very short sampling windows) (Supplemental Fig. S3) and explained future directions for making our model more complete.

In addition to our discovered entropy–sensitivity correlation, we have also expanded the strobemer family with mixedstrobes (combining *k*-mers and strobemers), altstrobes (alternated strobe lengths), and multistrobes (generalizing altstrobes). We experimentally verified that for most parameter settings in which they have higher entropy than randstrobes (the previously best-performing strobemer), they also produce higher seed sensitivity. We further validated the benefit of using mixedstrobes, altstrobes, and multistrobes as seeds using several metrics (Fig. 4; Supplemental Fig. S6; Sahlin 2021) and also showed that altstrobes and multistrobes have lower repetitiveness than, for example, randstrobes (Fig. 3B; Supplemental Fig. S10). Furthermore, we showed that mixedstrobes, altstrobes, and multistrobes are fast to construct (Fig. 8A,B; Supplemental Fig. S9) and do not constitute a bottleneck in mapping applications. We implemented randstrobes, mixedstrobes, and altstrobes in mininmap2 (Li 2018). Minimap2 uses subsampling of seeds, which distorts the relative entropies. Also, minimap2 implements chaining and other search-based cutoffs centered around minimizers. Nevertheless, we observed that using subsampled randstrobes and mixedstrobes within minimap2 for the most divergent sequence (10% mutation rates) both reduced runtime compared with *k*-mers of the same size with 25%–30% and resulted in 0.2% more correctly mapped reads on CHM13 (Supplemental Fig. S9). Finally, we estimated the ANI with *k*-mers and our new strobemer constructs. Our analysis indicated that higher entropy seeds are also better at estimating the relative rank of sequence's various error rates for both the simulated and ONT data (Figs. 6, 7, *R*^2^). However, as for estimating the actual ANI, *k*-mers performed the best, which is likely because the Mash distance was designed for *k*-mers. However, a modified Mash distance formula (Methods) fitted individually for *k*-mers, spaced *k*-mers, and strobemers (Methods) showed that the highest entropy strobemers both estimated the ANI best and achieved the best rank correlation. We believe that strobemers could be used for ANI estimation, particularly for ranking sequence similarity, which is used for selecting the best sequence out of multiple candidates and may be relevant for metagenomic sequence assignment. However, this requires the design of an ANI formula tailored for strobemers of various parameter settings.

We believe that our work opens up for future work in several directions. First, we may use our work's insights to produce even better seed constructs. For example, our model suggests that finding seed constructs with higher entropy could improve sensitivity further. Another example is that, guided by our results (for *k*_*s*_ = 1) (Fig. 2B), it seems viable to investigate multistrobes with different sampling distributions over sizes, such as only using a subset of strobe lengths, for example, three, seven, 11, 15, or similar. More generally, it remains to be explored what the maximal entropy of H(X|Y) is. We have not found a way to compute a reasonably tight upper bound as there is a nontrivial dependence between *X*_*i*_ and *Y*_*j*_. A seed with high entropy will have the *c* sampled positions scattered around in the *w* possible slots. A crude bound on H(X|Y) would be the entropy of *w* independent Bernoulli variables with *p* = *c*/*w*. The entropy for *w* independent Bernoulli variables, which has no guarantee that exactly *c* positions will be sampled, comes out to 63 for (30, 64)-seeds, 94 for (30, 114)-seeds, and 125 for (30, 214)-seeds, which are the parametrizations we investigated. However, we note that these values overestimate the true maximal value. A (*c, w*)-seed is restricted to sample exactly *c* out of *w* positions, lowering the entropy (https://math.stackexchange.com/q/2682723). It also remains to be explored whether such a randomly scattered seed construct can be designed in practice.

Second, we believe that incorporating probabilities of error-free runs (Blanca et al. 2022) will improve our model, which is currently only modeling entropy. Third, it is common to subsample of seeds to reduce memory footprint and processing time. We are interested in adapting our model to incorporate subsampling. It is clear that when subsampling, the advantage that pseudorandom seed constructs (e.g., strobemers) have over *k*-mers reduces (Supplemental Table S1; also shown by Sahlin 2021). This is because the high overlap of *k*-mers is removed with subsampling. Nevertheless, it would, for example, be beneficial to understand which subsampling densities and methods are suitable for pseudorandom seeds. Fourth, because the minimap2 implementation is centered around minimizers, it is possible that aligners customized for, for example, strobemers or other pseudorandom seeds may enjoy an even more substantial performance gain, as shown for short-read alignment (Sahlin 2022). Fifth, strobemers showed promising initial results for ANI estimation. It would be interesting to explore how strobemers can be used for ANI estimation in relevant applications. A first step is to find an ANI formula adjusted for strobemer seeds.

## Methods

### Formalizing entropy of seed constructs

#### Notation

We define a *subsequence* of a string as a set of ordered nucleotides that can be derived from the string by removing some or no elements while keeping the order of the remaining elements. If all letters in the subsequence are consecutive, we refer to it as a substring. We use zero-indexed notation for indexing sequences or strings, and we write *S*[*i*:*j*], *i* < *j* to refer to a substring of a string *S* starting at position *i* and ending at *j* but not including the character at *j*. That is, the start index is inclusive, but the end index is exclusive. We let the |⋅| operator denote the length of strings; for example, |S[i:j]|=j−i. We let the + operator denote string concatenation if applied to strings. Finally, we use *h* to denote a hash function mapping strings to integers.

### Strobemers

Strobemers are seeds that consist of *n* > 1 *ℓ*-mers (strobes) (Sahlin 2021). The first strobe *s*_1_ is the *ℓ*-mer at the position where the seed should be extracted. The subsequent strobes *s*_2_, …*s*_*n*_ are chosen from downstream windows defined by a lower (*w*_min_) and upper (*w*_max_) offset to its respective previous strobe's window. Hence, strobemers are characterized by the number of strobes (*n*), the length of the strobes (*ℓ*), and the window constraints (*w*_min_ and *w*_max_). Three methods to construct downstream strobes *s*_2_, …, *s*_*n*_ were given by Sahlin (2021): minstrobes, hybridstrobes, and randstrobes. For minstrobes, strobe *s*_2_ is simply the minimizer (Roberts et al. 2004) in the window [*w*_min_, *w*_max_] downstream from the first strobe. Hybridstrobes partition [*w*_min_, *w*_max_] into *x* subwindows and pick as *s*_2_ the minimizer in subwindow *h*(*s*_1_)%*x* (ordered 0 to *x* − 1). Randstrobes, the most effective seed by Sahlin (2021), select as *s*_2_ the *ℓ*-mer *s*′ in the window [*w*_min_, *w*_max_] that minimizes a hash function *h*(*s*_1_ + *s*′) (although there are variations to string concatenation) (see Sahlin 2021). Further downstream strobes if *n* > 2 are sampled analogously to *S*_2_. This study will mostly consider strobemers with *n* = 2.

#### Notation of seeds

For convenience, we use the following general notation for the construction of seeds. Let *c* and *w* be two positive integers with *c* ≤ *w*, where *c* denotes the number of distinct positions sampled in a substring of length *w* in a string *S*. Let *f*(*i*, *c*, *w*, *S*, *) denote some function that starts at position *i* in *S* and extracts a subsequence of characters at *c* distinct positions in the substring *S*[*i*:*i* + *w*] using only the information in *S*[*i*:*i* + *w*]. We use the final argument * to denote any seeding specific parameters that may include, for example, the sampling pattern for spaced *k*-mers or the parameters (*n*, *ℓ*, *w*_min_, *w*_max_) for strobemers. We will refer to seeds constructed by any *f* with parameters *c* and *w* as a (*c, w*)-seed.

#### Pseudorandom seeds

For the seed to be a pseudorandom seed, *f* needs to use a pseudorandom sampling decision. Seeds such as *k*-mers and spaced *k*-mers have a fixed sampling pattern and are not pseudorandom seeds. The pseudorandomness in strobemer seeds is enforced through the hash function *h*, which decides which strobes to sample. The selection of subsequent strobes *s*_2_, …*s*_*n*_ appears random but is fully deterministic given a hash function and the underlying sequence from which seeds are sampled.

#### Modeling entropy of a pseudorandom seed

We define entropy of a seed as follows. Consider a position *p* on a string *S* with *w* − 1 ≤ *p* ≤ |*S*| − *w*; that is, *p* is not close to the boundaries. For any given seed, let *Y*_*j*_ ∈ 0, 1 be the binary variable denoting if position *j* ∈ [0, *k* − 1] is sampled from *p* on *S* (*Y*_*j*_ = 1). Let *i* be an indexing integer on the reference starting at *i* = 0 for the position immediately downstream from *p*, and *X*_*i*_ be the binary variable describing the event that position *i* is sampled by the same seed (Fig. 1B). Then $X_{i}|\;Y_{j}$ describes the event that position *i* is sampled on *S* conditioned on that position *p* in *S* has been sampled at position *j* in the seed, and $P(X_{i}|\;Y_{j})$ denotes the probability of this event. The conditional entropy of a seed sampling any downstream position given that *p* was sampled by the seed, X|Y, is computed as

(1) $$\begin{array}{lll} H(X|\;Y) & = & -\overset{k-1}{\underset{\;j=0}{\sum }}\;\overset{w-2}{\underset{i=0}{\sum }}\;P(Y_{j})P(X_{i}|\;Y_{j})\log_{2}\;P(X_{i}|\;Y_{j})\\ & = & -\frac{1}{k}\overset{k-1}{\underset{\;j=0}{\sum }}\;\overset{w-2}{\underset{i=0}{\sum }}\;P(X_{i}|\;Y_{j})\log_{2}\;P(X_{i}|\;Y_{j}). \end{array}$$

Here *P*(*Y*_*j*_) = 1/*k* by the assumption that any position *j* on the seed is equally likely to be sampled from reference position *p* if we pick a position *p* at random. Also, variable *i* only needs to be summed up to *w* − 2 as positions further downstream will have a probability of zero. The probability $P(X_{i}|\;Y_{j})$ is specific to the seed construct but can, for strobemers, be structured up into cases and is relatively straightforward to compute. We provide example computations for *k*-mers, randstrobes, altstrobes, mixedstrobes, and multistrobes in Supplemental Section S2. All seed constructs without pseudorandomness such as *k*-mers and spaced *k*-mers have an entropy of zero according to Equation 2, as $P(X_{i}|\;Y_{j})$ is either zero or one.

Our entropy measure cannot estimate entropy for seed constructs that pass information (are correlated) between neighboring seeds for pseudorandom decisions. An example of such a seed construct is hybridstrobes, which use minimizers (Roberts et al. 2004) that can be shared between neighboring windows. Nevertheless, we will see that the estimate will be a useful predictor for randstrobes and other pseudorandom seed constructs that we introduce in this study. Finally, our model is agnostic to the underlying error pattern, for example, the relative fraction of substitutions to indels. It is known that some patterns such as spaced *k*-mers perform well when substitutions are more frequent than indels (Leinonen and Salmela 2022).

### Evaluating seed performance

#### Constraints on f

We impose the following three basic constraints on *f* to be viable for sequence matching.

C1. *f* produces the same (*c, w*)-seeds for two strings *S* and *T* if *S* = *T*.
C2. *f* produces valid (*c, w*)-seeds $\forall S,S\in \Sigma^{*}$.
C3. At most one seed is produced per position in a sequence.

C1 and C2 are necessary for sequence matching. An example of a construct that violates C2 is “sample the position if the letter is A or C” because there may not be enough A's and C's in the window. C3 limits querying to, at most, one lookup per position, making the constructs efficient. We have intentionally described *f* in a general fashion in order to encompass more general construction techniques. For example, a *k*-mer would deterministically sample the first *k* nucleotides (nt), regardless of the size of *w*. A randstrobe (Sahlin 2021) with parameters (2,15,25,50) is a valid (30, 64)-seed because the maximum span of sampled positions in the randstrobe is *w* = 50 + 15 − 1 = 64, and *f* would sample 15 nt at *S*[*i*:*i* + 15] as the first strobe and then sample the next strobe starting somewhere in *S*[*i* + 25:*i* + 50].

#### E-hits of seeds

We will in our assessment of seeds need a notion of seed repetitiveness, and we will use E-hits for this. The definition of E-hits was given by Sahlin (2022) and is a measure of how repetitive the seeds in a query sequence are, on average, in a reference data set. More specifically, E-hits compute the expected number of hits that a seed matched to the reference will have given that the seed is error-free and comes from a query uniformly sampled from the reference. E-hits can be calculated for any seeding mechanism and reference data set. For a given reference data set, let *N* be the total number of seeds sampled, *M* the total number of distinct seeds sampled, and *z*_*i*_ be the total number of times the distinct seed *i* (1 ≤ *i* ≤ *M*) is sampled. Then, E-hits is computed as follows:

(2) $$E-\mathrm{hits}=\frac{1}{N}\overset{M}{\underset{i=1}{\sum }}\;z_{i}^{2}.$$

#### Objectives for sequence similarity detection

Our objective is to maximize the probability that a seed matches between two homologous sequences, given any number of mutations, while remaining precise enough not to yield spurious matches to nonhomologous regions. We state the objectives in precise terms here. Let two strings *S* and *T*, each of length 2*w*, have an edit distance *m* ≥ 0 to each other. Let *N*_*m*_(*c*, *w*) be the number of seed matches from the first *w* consecutive (*c, w*)-seeds constructed from *S* and *T* (see Fig. 1A). We desire a function *f* that extract (*c, w*)-seeds such that

O1. *P*(*N*_*m*_(*c*, *w*) > 0) is as large as possible ∀m≥0.
O2. The E-hits metric (Sahlin 2022) for *f* is as small as possible.

O1 relates to seed sensitivity, and O2 relates to seed repetitiveness. The formulation of *N*_*m*_(*c*, *w*), namely, to only consider the first *w* seeds in a region of 2*w* for short strings, may seem unfair to *k*-mers. This is because *k*-mers can produce additional hits between *S* and *T* from the last *w* − *k* seeds at the ends of *S* and *T* (see Fig. 1A). This advantage is present at the end of each sequence or at the end of every alignment site in the case of split alignments by large indels or rearrangements. We aim to model a scenario in which sequences are substantially longer than the extra *w* − *k* seeds in the ends, for example, as for long reads. Therefore, O1 reflects all regions but the *w*-long end region of sequences.

### Alternative strobemer constructs

Based on our intuition that randomness would improve sensitivity and our designed model for estimating entropy, we wanted to explore whether altering various parameters in the original strobemer constructs (proposed by Sahlin 2021) would lead to higher entropy and, therefore, higher sensitivity. We here propose three alternative seed constructs to the strobemer seed-family: mixedstrobes, altstrobes, and multistrobes. We will later show that these seed constructs, for some parametrizations, can yield higher sensitivity than randstrobes, which was the most sensitive seed proposed by Sahlin (2021). Notably, the parametrizations that receive higher sensitivity than randstrobes also receive higher entropy in our model, although the reverse is not always true.

#### Mixedstrobes

Mixedstrobes samples either a *k*-mer or a strobemer at a specified fraction. Any strobemer may be sampled, but we will only consider randstrobes here. We parameterize mixedstrobes as (*n*, *ℓ*, *w*_min_, *w*_max_, *q*), where *n* is the number of strobes, *ℓ* is the strobe length, *w*_min_ and *w*_max_ are the minimum and maximum downstream offsets to last window, and *q* is the strobemer fraction. Whether a strobemer or a *k*-mer is seeded depends on the hash value of the first strobe *h*(*S*[*i*:*i* + *ℓ*]) and the user-defined strobe fraction *q*. The strobe fraction *q* is represented as numerator *N* and a denominator *D* (e.g., *q* = 0.6 is represented as *N* = 60 and *D* = 100) so that

$$f(i,k,w,S,*)=\{\begin{array}{ll} \mathrm{Sample}\;\mathrm{strobemer},\;\; & \mathrm{if}\;h(S[i:i+\ell ])\;\text{\%}\;D<N\;\\ \mathrm{Sample}\;k-\mathrm{mer},\;\; & \mathrm{otherwise}\; \end{array}$$

The full pseudocode to construct mixedstrobes is given in Algorithm 1 in Supplemental Section S1.

#### Altstrobes

Altstrobes are modified randstrobes in which the strobe length alternates (hence, altstrobes) between shorter and longer strobes. For example, instead of having two strobes of length *k*/2 as implemented in randstrobes of order 2, altstrobes of order 2 consist of one short strobe *k*_*s*_ and one longer strobe *k*_*l*_, with |*k*_*s*_| + |*k*_*l*_| = *k*. We parameterize altstrobes as (*n*, |*k*_*s*_|, |*k*_*l*_|, *w*_min_, *w*_max_). We refer to sampled altstrobes with *n* = 2 as (|*k*_*s*_|, |*k*_*l*_|) or (|*k*_*l*_|, |*k*_*s*_|), depending on if the short strobe was used first or second, respectively. We decide the length of the first strobe based on the hash value of the substring of length |*k*_*s*_| (i.e., the potential first strobe). Specifically,

$$\begin{array}{ll} & f(i,k,w,S,*)=\\ & \{\begin{array}{ll} \mathrm{Sample}\;\mathrm{altstrobe}\;(k_{s},k_{l}),\;\; & \mathrm{if}\;h(S[i:i+|k_{s}|])\;\text{\%}\;2=0\;\\ \mathrm{Sample}\;\mathrm{altstrobe}\;(k_{l},k_{s}),\;\; & \mathrm{otherwise}\; \end{array} \end{array}$$

The sampled strobe length is decided by the hash value of the shorter strobe. Otherwise, mutations within the positions [*k*_*s*_, *k*_*l*_] downstream from the start position may lead to seeds being sampled differently between two sequences, which leads to unnecessary seed mismatches. The sampling of the second strobe is performed identically to randstrobes in a downstream window specified by *w*_min_ and *w*_max_ as described by Sahlin (2021).

For fair benchmarking to other strobemer seeds, we implement two evaluation-specific constraints on altstrobes. First, *n* has to be even to guarantee seeds with the same number of sampled positions. Second, to guarantee that all altstrobe seeds are (*c, w*)-seeds, we adjust the sampling window offset depending on if it is the long or short strobe we sample first. Specifically, we let *k*_*l*_ in altstrobe (*k*_*s*_, *k*_*l*_) be sampled from [*w*_min_ − (*k*_*l*_ − *k*_*s*_)/2, *w*_max_ − (*k*_*l*_ − *k*_*s*_)/2] and *k*_*s*_ in altstrobe (*k*_*l*_, *k*_*s*_) be sampled from [*w*_min_ + (*k*_*l*_ − *k*_*s*_)/2, *w*_max_ + (*k*_*l*_ − *k*_*s*_)/2]. These constraints are only implemented for controlled benchmarking. The full pseudocode to construct altstrobes is given in Algorithm 2 in the Supplemental Section S1.

#### Multistrobes

Multistrobes are generalized altstrobes in which strobe lengths are selected in a range of lengths. They are parameterized identically to altstrobes as (*n*, |*k*_*s*_|, |*k*_*l*_|, *w*_*min*_, *w*_*max*_) with |*k*_*s*_| + |*k*_*l*_| = *k*. However, unlike altstrobes, they can sample any length of a strobe in [|*k*_*s*_|, |*k*_*l*_|]. Similar to but more generally expressed than altstrobes, the strobe length of the first sampled strobe *k*_1_ in multistrobes is given by $\left|k_{1}\left|\;\overset{.}{=}\;\right|k_{s}\right|+(h(S[i:i+|k_{s}|])\text{\%}(|k_{l}|-|k_{s}|+1))$. The second strobe length is then given the length $\left|k_{2}\left|\;\overset{.}{=}k-\right|k_{1}\right|$ and is sampled identically to altstrobes in a downstream window. We use the same evaluation-specific constraints as described for altstrobes for fair benchmarking to other strobemers.

For small *k*_*s*_, uniform sampling of lengths is not possible. For example, for |*k*_*s*_| = 1, only four possible hash values can be produced, which may be smaller than |*k*_*l*_ − *k*_*s*_ + 1|. Similar but not as extreme effects are present for |*k*_*s*_| 2 and 3, especially if several of the few possible hash values map to the same lengths. We use some implementation tricks to keep a high uniformity despite low *k*_*s*_ given in Supplemental Section S1.3. The full pseudocode to construct multistrobes is given in Algorithm 3 in Supplemental Materials.

### ANI estimation

#### Mash distance

The Mash distance is computed as

(3) $$I(A,B)/100=1+\frac{1}{k}\times \ln\;\left(\frac{2\cdot J(A,B)}{1+J(A,B)}\right),$$

where *J*(*A*, *B*) denotes the Jaccard distance between two (seed) sets *A* and *B*.

#### Modified Mash distance

Using the original Mash distance, we did not observe as good agreement (TSS) between the estimated ANI (Mash distance) and the true ANI as for *k*-mers. This is expected, as the Mash distance is specifically designed for *k*-mers. Therefore, we introduced a correction term cf*(1 − *I*(*A*, *B*)) and fitted the correction factor individually for *k*-mers, spaced *k*-mers, and strobemers with

(4) I(A,B)′=I(A,B)+cf∗(1−I(A,B)).

We selected the best possible correction factor fit for each of the methods. That is, *cf* was set to the value that resulted in the lowest TSS for each of the methods, which were 0.075, 0.165, 0.3, and 0.6 for *k*-mers, strobemers, and dense- and sparse-spaced *k*-mers, respectively. Note, that the correction term does not alter the rank correlation (*R*^2^) metric.

#### Simulated experiments

For our simulated experiments, we sampled 1000 random reference sequences of 1000 nt, whereby the probability of each of the 4 nt was 25% for each position. We then simulated corresponding query sequences (reads) for each experimental condition by introducing mutations to the reference sequence, with the percentage of mutated nucleotides ranging from 0.5% to 10% in increments of 0.5%, yielding 20,000 benchmarking sequences. Insertions, deletions, and substitutions were hereby added with equal probability of 1/3, which represent typical error profiles in ONT reads (Sahlin and Medvedev 2021).

Next, we constructed seeds from the sequences with *k*-mers, (*k* = 30), spaced *k*-mers with different two different densities, and strobemers (2, 15, 25, 50) and subsequently computed the Mash distance for each sequence. If a method did not yield any matches for a certain sequence, we did not compute the Mash distance of it and discarded it from the analysis (<0.1% of *k*-mers and minstrobes, <0.25% of dense-spaced *k*-mers, and ∼20% of sparse-spaced *k*-mers). No read was discarded for the other strobemer methods (hybridstrobes, randstrobes, mixedstrobes, altstrobes, multistrobes). The high number of spaced *k*-mers discarded is owing to the high indel rate in the reads with the highest error rate.

#### ONT experiments

To estimate the ANI for *Escherichia coli* Oxford Nanopore Technology reads, we first computed the longest collinear chain of raw hits for each disjoint segment of 2000 bp to obtain the number of matches while avoiding overcounting spurious hits (for details, see Supplemental Sec. 4.4). Based on the number of matching and nonmatching seeds, we computed the adjusted Mash distance provided above.

The adjusted Mash distances of all segments from all reads were combined (mean) to estimate the ANI. As we do not know the true mutation rate of the reads, we computed the read-to-genome distance using the online GGDC 3.0 with default settings (Meier-Kolthoff et al. 2013, 2022) and compared the ANI estimates of each segment to the GGDC score using the TSS adjusted to equal sample size.

### Data analysis

Data analysis was conducted in R 4.1.3 (R Core Team 2022) and Python 3.10 (Van Rossum and Drake 2009). Figures were produced using the packages ggplot2 3.3.5(Wickham 2016) and ggh4x 0.2.3 (https://github.com/teunbrand/ggh4x, https://teunbrand.github.io/ggh4x/) in tidyverse 1.3.1 (R) (Wickham et al. 2019) and Matplotlib 3.5.1 (Hunter 2007) and seaborn 0.11.2 (Python) (Waskom 2021).

### Software availability

All the scripts used for the analysis and evaluation, as well as our seed implementations in StrobeMap and minimap2, are available at GitHub (https://github.com/benjamindominikmaier/mixedstrobes_altstrobes) and as Supplemental Code.

## Supplementary Material

Supplement 1

Supplement 2
