# Supplementary material for: Entropy predicts sensitivity of pseudorandom seeds
Source: Genome Res. 2023 Jul;33(7):1162–74. doi: 10.1101/gr.277645.123 (PMC10538493; doi:10.1101/gr.277645.123)
Supplement: Supplement 1 [file Supplemental_code.zip › src/figures.html]

figures.knit


#!/usr/bin/Rscript

SCRIPT NAME: figures.rmd  
PURPOSE: Generate figures for Maier & Sahlin, 2023 (bioRxiv
2022.10.13.512198)  
—————————  
AUTHOR: Benjamin Dominik Maier  
CREDITS: Benjamin D. Maier (0000-0001-8442-0536) & Kristoffer Sahlin
(0000−0001−7378−2320)  
ORGANIZATION: Department of Mathematics, Science for Life Laboratory,
Stockholm University, 106 91, Stockholm, Sweden.  
EMAIL: kristoffer.sahlin [at] scilifelab.se  
—————————  
DATE CREATED: 01.02.2022  
LAST MODIFIED: 11.03.2023  
—————————

## Loading Packages

```
library("tidyverse")
```

```
## ── Attaching packages ─────────────────────────────────────── tidyverse 1.3.2 ──
## ✔ ggplot2 3.4.1     ✔ purrr   1.0.1
## ✔ tibble  3.1.8     ✔ dplyr   1.1.0
## ✔ tidyr   1.3.0     ✔ stringr 1.5.0
## ✔ readr   2.1.4     ✔ forcats 1.0.0
## ── Conflicts ────────────────────────────────────────── tidyverse_conflicts() ──
## ✖ dplyr::filter() masks stats::filter()
## ✖ dplyr::lag()    masks stats::lag()
```

```
library(viridis)
```

```
## Loading required package: viridisLite
```

```
library(scales)
```

```
## 
## Attaching package: 'scales'
## 
## The following object is masked from 'package:viridis':
## 
##     viridis_pal
## 
## The following object is masked from 'package:purrr':
## 
##     discard
## 
## The following object is masked from 'package:readr':
## 
##     col_factor
```

```
library(ggh4x)
sessionInfo()
```

```
## R version 4.2.2 Patched (2022-11-10 r83330)
## Platform: x86_64-pc-linux-gnu (64-bit)
## Running under: Ubuntu 22.10
## 
## Matrix products: default
## BLAS:   /usr/lib/x86_64-linux-gnu/blas/libblas.so.3.10.1
## LAPACK: /usr/lib/x86_64-linux-gnu/lapack/liblapack.so.3.10.1
## 
## locale:
##  [1] LC_CTYPE=en_US.UTF-8       LC_NUMERIC=C              
##  [3] LC_TIME=de_DE.UTF-8        LC_COLLATE=en_US.UTF-8    
##  [5] LC_MONETARY=de_DE.UTF-8    LC_MESSAGES=en_US.UTF-8   
##  [7] LC_PAPER=de_DE.UTF-8       LC_NAME=C                 
##  [9] LC_ADDRESS=C               LC_TELEPHONE=C            
## [11] LC_MEASUREMENT=de_DE.UTF-8 LC_IDENTIFICATION=C       
## 
## attached base packages:
## [1] stats     graphics  grDevices utils     datasets  methods   base     
## 
## other attached packages:
##  [1] ggh4x_0.2.3       scales_1.2.1      viridis_0.6.2     viridisLite_0.4.1
##  [5] forcats_1.0.0     stringr_1.5.0     dplyr_1.1.0       purrr_1.0.1      
##  [9] readr_2.1.4       tidyr_1.3.0       tibble_3.1.8      ggplot2_3.4.1    
## [13] tidyverse_1.3.2  
## 
## loaded via a namespace (and not attached):
##  [1] tidyselect_1.2.0    xfun_0.37           bslib_0.4.2        
##  [4] haven_2.5.1         gargle_1.3.0        colorspace_2.1-0   
##  [7] vctrs_0.5.2         generics_0.1.3      htmltools_0.5.4    
## [10] yaml_2.3.7          utf8_1.2.3          rlang_1.0.6        
## [13] jquerylib_0.1.4     pillar_1.8.1        withr_2.5.0        
## [16] glue_1.6.2          DBI_1.1.3           dbplyr_2.3.0       
## [19] modelr_0.1.10       readxl_1.4.2        lifecycle_1.0.3    
## [22] munsell_0.5.0       gtable_0.3.1        cellranger_1.1.0   
## [25] rvest_1.0.3         evaluate_0.20       knitr_1.42         
## [28] tzdb_0.3.0          fastmap_1.1.0       fansi_1.0.4        
## [31] broom_1.0.3         backports_1.4.1     googlesheets4_1.0.1
## [34] cachem_1.0.5        jsonlite_1.8.4      fs_1.6.1           
## [37] gridExtra_2.3       hms_1.1.2           digest_0.6.31      
## [40] stringi_1.7.12      grid_4.2.2          cli_3.6.0          
## [43] tools_4.2.2         magrittr_2.0.3      sass_0.4.0         
## [46] crayon_1.5.2        pkgconfig_2.0.3     ellipsis_0.3.2     
## [49] xml2_1.3.3          reprex_2.0.2        googledrive_2.0.0  
## [52] lubridate_1.9.2     timechange_0.2.0    assertthat_0.2.1   
## [55] rmarkdown_2.20      httr_1.4.4          rstudioapi_0.14    
## [58] R6_2.5.1            compiler_4.2.2
```

## Create Folder Structure

```
ifelse(!dir.exists(paths = "../output/figs"), dir.create(file.path("../output/figs")), FALSE)
```

```
## [1] FALSE
```

```
folder_names <- c("ANI",
                  "benchmarking_minimap", 
                  "benchmarking_strobemap",
                  "entropy_pmatch",
                  "matching_analysis_bio",
                  "matching_analysis_sim",
                  "uniqueness_esize")

for (folder_name in folder_names){
  ifelse(!dir.exists(paths = paste("../output/figs/", folder_name, sep="")),
       dir.create(file.path(paste("../output/figs/", folder_name, sep=""))),
       FALSE)
}
```

## Sampling of kmers, strobemers and altstrobes

```
knitr::include_graphics("../output/figs/figure1.pdf")
```

## Empirically verifying correlation between seed entropy and sensitivity

```
results_entropy_all <- rbind(
  read.table("../output/data tables/p_match/10000_30_16_50_pypy.csv", header=T, sep=","),
  read.table("../output/data tables/p_match/10000_30_25_50_pypy.csv", header=T, sep=","),
  read.table("../output/data tables/p_match/10000_30_35_50_pypy.csv", header=T, sep=","),
  read.table("../output/data tables/p_match/10000_30_45_50_pypy.csv", header=T, sep=","),
  #read.table("../output/data tables/p_match/10000_30_49_50_pypy.csv", header=T, sep=","),
  read.table("../output/data tables/p_match/1000_30_16_100_pypy.csv", header=T, sep=","),
  read.table("../output/data tables/p_match/1000_30_25_100_pypy.csv", header=T, sep=","),
  read.table("../output/data tables/p_match/1000_30_50_100_pypy.csv", header=T, sep=","),
  read.table("../output/data tables/p_match/1000_30_75_100_pypy.csv", header=T, sep=","),
  read.table("../output/data tables/p_match/1000_30_95_100_pypy.csv", header=T, sep=","),
  #read.table("../output/data tables/p_match/1000_30_99_100_pypy.csv", header=T, sep=","),
  read.table("../output/data tables/p_match/1000_30_16_200_pypy.csv", header=T, sep=","),
  read.table("../output/data tables/p_match/1000_30_50_200_pypy.csv", header=T, sep=","),
  read.table("../output/data tables/p_match/1000_30_100_200_pypy.csv", header=T, sep=","),
  read.table("../output/data tables/p_match/1000_30_150_200_pypy.csv", header=T, sep=","),
  read.table("../output/data tables/p_match/1000_30_195_200_pypy.csv", header=T, sep=",")) %>% 
  #read.table("../output/data tables/p_match/1000_30_199_200_pypy.csv", header=T, sep=",")) %>% 
  mutate(type = ifelse(type == "mixed-randstrobes", "mixedstrobes", type),
         type = ifelse(type == "continous altstrobes", "multistrobes", type),
         window = paste("W = [", w_min, ",", w_max, "]", sep = ""),
         w_max = paste("w_max = ", w_max, sep="")) %>% 
  filter(type != "Optimal")


results_entropy_all$w_min <- factor(results_entropy_all$w_min, levels = c(16, 25, 35, 45, 49, 50, 75, 95, 99, 100, 150, 195, 199))
#results_entropy_all$type <- factor(results_entropy_all$type, levels= c("Optimal", "k-mers", "altstrobes", "randstrobes", "mixedstrobes")) 
results_entropy_all$type <- factor(results_entropy_all$type, levels= c("k-mers", "altstrobes", "randstrobes", "mixedstrobes", "multistrobes"))
results_entropy_all$window <- factor(results_entropy_all$window, levels = c("W = [16,50]", "W = [25,50]", "W = [35,50]", "W = [45,50]", "W = [49,50]", "W = [16,100]", "W = [25,100]", "W = [50,100]", "W = [75,100]", "W = [95,100]", "W = [99,100]", "W = [16,200]", "W = [50,200]", "W = [100,200]", "W = [150,200]", "W = [195,200]", "W = [199,200]"))
results_entropy_all$w_max <- factor(results_entropy_all$w_max, levels = c("w_max = 50", "w_max = 100", "w_max = 200"))
results_entropy_all <- results_entropy_all %>% mutate(x = ifelse(x == 30, 0, x))


results_entropy_scatter <- full_join(
  results_entropy_all %>% filter(ymetric == "Entropy") %>% rename('entropy' = 'y') %>% select(w_min, w_max, window, type, x, entropy),
  results_entropy_all %>% filter(ymetric == "p_match") %>% rename('sensitivity' = 'y') %>% select(w_min, w_max, window, type, x, sensitivity),
  by = c("w_min", "w_max", "window", "type", "x"))
```

```
## Warning in full_join(results_entropy_all %>% filter(ymetric == "Entropy") %>% : Each row in `x` is expected to match at most 1 row in `y`.
## ℹ Row 1 of `x` matches multiple rows.
## ℹ If multiple matches are expected, set `multiple = "all"` to silence this
##   warning.
```

```
results_entropy_scatter$type <- factor(results_entropy_scatter$type, levels= c("k-mers", "altstrobes", "randstrobes", "mixedstrobes", "multistrobes"))
results_entropy_all$window <- factor(results_entropy_all$window, levels = c("W = [16,50]", "W = [25,50]", "W = [35,50]", "W = [45,50]", "W = [49,50]", "W = [16,100]", "W = [25,100]", "W = [50,100]", "W = [75,100]", "W = [95,100]", "W = [99,100]", "W = [16,200]", "W = [50,200]", "W = [100,200]", "W = [150,200]", "W = [195,200]", "W = [199,200]"))

pearson_correlation <- results_entropy_scatter %>% group_by(w_min, w_max, window) %>% summarise(pearson = cor(entropy, sensitivity, method = "pearson"), min_ent = min(entropy), max_sens = max(sensitivity)) %>% mutate(R2 = round(pearson*pearson, 2))
```

```
## `summarise()` has grouped output by 'w_min', 'w_max'. You can override using
## the `.groups` argument.
```

```
results_entropy_scatter <- results_entropy_scatter %>% 
  left_join(pearson_correlation, by = c("w_min", "w_max", "window"))

design <- "ABCDE
           FGHIJ"
design_full <- "ABCD#
                EFGHI
                JKLMN"
```

```
ggplot(results_entropy_scatter %>% filter(w_max == "w_max = 50"))+
  #geom_point(aes(x = entropy, y = sensitivity, col= type, shape=w_min))+
  geom_point(aes(x = entropy, y = sensitivity, col= type))+
  geom_text(aes(x= 1, y=13, hjust = 0, label=paste("R² = ", R2, sep = "")))+
  scale_color_manual(values = c("#F8766D", "#E76BF3", "#00B0F6", "blue", "black"))+
  theme(legend.position = "none")+
  xlim(0, 30)+ ylim(9.75, 13)+
  facet_wrap(~window, nrow = 1)
```

```
## Warning: Removed 10 rows containing missing values (`geom_point()`).
```

```
ggsave("../output/figs/entropy_pmatch/fig_2A_sens_entr.pdf", width = 7, height = 2)
```

```
## Warning: Removed 10 rows containing missing values (`geom_point()`).
```

```
ggsave("../output/figs/entropy_pmatch/fig_2A_sens_entr.png", width = 7, height = 2)
```

```
## Warning: Removed 10 rows containing missing values (`geom_point()`).
```

```
#ggplot(results_entropy_scatter %>% filter(w_min == 25 & w_max == "w_max = 50"))+
ggplot(results_entropy_scatter %>% filter(w_max != "w_max = 50"))+
  #geom_point(aes(x = entropy, y = sensitivity, col= type, shape=w_min))+
  geom_point(aes(x = entropy, y = sensitivity, col= type))+
  geom_text(aes(x= 1, y=1.01*max_sens, hjust = 0, label=paste("R² = ", R2, sep = "")))+
  scale_color_manual(values = c("#F8766D", "#E76BF3", "#00B0F6", "blue", "black"))+
  theme(legend.position = "none")+
  facet_manual(~window, scales = "free", design = design)
```

```
ggsave("../output/figs/entropy_pmatch/suppl_fig_1_sens_entr.pdf", width = 7, height = 3.75)
ggsave("../output/figs/entropy_pmatch/suppl_fig_1_sens_entr.png", width = 7, height = 3.75)


results_entropy_scatter$window <- factor(results_entropy_scatter$window, levels = c("W = [16,50]", "W = [25,50]", "W = [35,50]", "W = [45,50]", "W = [49,50]", "W = [16,100]", "W = [25,100]", "W = [50,100]", "W = [75,100]", "W = [95,100]", "W = [99,100]", "W = [16,200]", "W = [50,200]", "W = [100,200]", "W = [150,200]", "W = [195,200]", "W = [199,200]"))
#ggplot(results_entropy_scatter %>% filter(w_min == 25 & w_max == "w_max = 50"))+
ggplot(results_entropy_scatter)+
  #geom_point(aes(x = entropy, y = sensitivity, col= type, shape=w_min))+
  geom_point(aes(x = entropy, y = sensitivity, col= type))+
  geom_text(aes(x= 1, y=1.025*max_sens, hjust = 0, label=paste("R² = ", R2, sep = "")))+
  geom_text(aes(x= 1, y=1.05*max_sens, hjust = 0, label=paste("")))+
  scale_color_manual(values = c("#F8766D", "#E76BF3", "#00B0F6", "blue", "black"))+
  theme(legend.position = "none")+
  facet_manual(~window, scales = "free", design = design_full)
```

```
ggsave("../output/figs/entropy_pmatch/additional_sens_entr_full.pdf", width = 7, height = 5)
ggsave("../output/figs/entropy_pmatch/additional_sens_entr_full.png", width = 7, height = 5)
```

```
ggplot(results_entropy_all %>% filter(w_max == "w_max = 50") %>% mutate(ymetric = ifelse(ymetric == "p_match", "Sensitivity", ymetric)), aes(group = interaction(w_min,type)))+
  geom_line(aes(x = x, y = y, col = type), size=0.5)+
  geom_point(aes(x = x, y = y, fill = type, shape = w_min), size = 2, stroke = 0.25)+
  theme(axis.text.x = element_text(angle = 90, vjust = 0.5, hjust=1),
        strip.text.x = element_text(size = 11),
        strip.text.y = element_text(size = 11),
        legend.position = "bottom",
        legend.box="vertical",
        legend.title=element_blank(),
        legend.text = element_text(size = 11),
        legend.margin=margin())+
  labs(x = "Length k[s]               Fraction Strobemers             Length k[s]",
       y = "")+
  scale_fill_manual(values = c("#F8766D", "#E76BF3", "#00B0F6", "blue", "black"))+
  scale_color_manual(values = c("#F8766D", "#E76BF3", "#00B0F6", "blue", "black"))+
  scale_shape_manual(values = c(21,24,23,22,25))+
  facet_nested(w_max+ymetric ~ analysis, scales = "free")+
  expand_limits(x = 0)+
  guides(shape = guide_legend(order = 2),col = guide_legend(order = 1), size = "none", linetype = "none", fill = "none")
```

```
## Warning: Using `size` aesthetic for lines was deprecated in ggplot2 3.4.0.
## ℹ Please use `linewidth` instead.
```

```
ggsave("../output/figs/entropy_pmatch/fig_2b_entropy_pmatch_50.png", width = 6, height = 4.5)
ggsave("../output/figs/entropy_pmatch/fig_2b_entropy_pmatch_50.pdf", width = 6, height = 4.5)
ggsave("../output/figs/entropy_pmatch/fig_2b__entropy_pmatch50_RECOMB.pdf", width = 6, height = 4.0)

ggplot(results_entropy_all %>% filter(ymetric == "Entropy" & w_max == "w_max = 50"), aes(group = interaction(w_min,type)))+
  geom_line(aes(x = x, y = y, col = type), size=0.25)+
  geom_point(aes(x = x, y = y, fill = type, shape = w_min), size = 2, stroke = 0.25)+
  theme(axis.text.x = element_text(angle = 90, vjust = 0.5, hjust=1),
        strip.text.x = element_text(size = 11),
        strip.text.y = element_text(size = 11),
        legend.position = "none",
        legend.box="vertical",
        legend.title=element_blank(),
        legend.text = element_text(size = 11),
        legend.margin=margin())+
  labs(x = "Length k[s]                Fraction Strobemers                Length k[s]",
       y = "Entropy")+
  scale_color_manual(values = c("#F8766D", "#E76BF3", "#00B0F6", "blue", "black"))+
  scale_fill_manual(values = c("#F8766D", "#E76BF3", "#00B0F6", "blue", "black"))+
  scale_size_manual(values = c(1,1,1,1,1))+
  scale_shape_manual(values = c(21,24,23,22,25,18))+
  facet_grid(w_max ~ analysis, scales = "free")+
  expand_limits(x = 0, y = 0)+
  guides(shape = guide_legend(order = 2),col = guide_legend(order = 1), size = "none", linetype = "none", fill = "none")
```

```
ggsave("../output/figs/entropy_pmatch/additional_entropy_50.png", width = 5.75, height = 2.5)
ggsave("../output/figs/entropy_pmatch/additional_entropy_50.pdf", width = 5.75, height = 2.5)

ggplot(results_entropy_all %>% filter(ymetric == "p_match" & w_max == "w_max = 50"), aes(group = interaction(w_min,type)))+
  geom_line(aes(x = x, y = y, col = type), size=0.25)+
  geom_point(aes(x = x, y = y, fill = type, shape = w_min), size = 2, stroke = 0.25)+
  theme(axis.text.x = element_text(angle = 90, vjust = 0.5, hjust=1),
        strip.text.x = element_text(size = 11),
        strip.text.y = element_text(size = 11),
        legend.position = "none",
        legend.box="vertical",
        legend.title=element_blank(),
        legend.text = element_text(size = 11),
        legend.margin=margin())+
  labs(x = "Length k[s]                Fraction Strobemers               Length k[s]",
       y = "Sensitivity")+
  scale_fill_manual(values = c("#F8766D", "#E76BF3", "#00B0F6", "blue", "black"))+
  scale_color_manual(values = c("#F8766D", "#E76BF3", "#00B0F6", "blue", "black"))+
  scale_shape_manual(values = c(21,24,23,22,25))+
  facet_grid(w_max ~ analysis, scales = "free")+
  expand_limits(x = 0)+
  guides(shape = guide_legend(order = 2),col = guide_legend(order = 1), size = "none", linetype = "none", fill = "none")
```

```
ggsave("../output/figs/entropy_pmatch/additional_p_match_50.png", width = 5.75, height = 2.5)
ggsave("../output/figs/entropy_pmatch/additional_p_match_50.pdf", width = 5.75, height = 2.5)
```

```
ggplot(results_entropy_all %>% filter(w_max != "w_max = 50") %>% mutate(ymetric = ifelse(ymetric == "p_match", "Sensitivity", ymetric)), aes(group = interaction(w_min,type)))+
  geom_line(aes(x = x, y = y, col = type), size=0.5)+
  geom_point(aes(x = x, y = y, fill = type, shape = w_min), size = 2, stroke = 0.25)+
  theme(axis.text.x = element_text(angle = 90, vjust = 0.5, hjust=1),
        strip.text.x = element_text(size = 11),
        strip.text.y = element_text(size = 11),
        legend.position = "bottom",
        legend.box="vertical",
        legend.title=element_blank(),
        legend.text = element_text(size = 11),
        legend.margin=margin())+
  labs(x = "Length k[s]               Fraction Strobemers             Length k[s]",
       y = "")+
  scale_fill_manual(values = c("#F8766D", "#E76BF3", "#00B0F6", "blue", "black"))+
  scale_color_manual(values = c("#F8766D", "#E76BF3", "#00B0F6", "blue", "black"))+
  scale_shape_manual(values = c(21,24,23,22,25,7,8,9))+
  facet_nested(w_max+ymetric ~ analysis, scales = "free")+
  expand_limits(x = 0)+
  guides(shape = guide_legend(order = 2),col = guide_legend(order = 1), size = "none", linetype = "none", fill = "none")
```

```
ggsave("../output/figs/entropy_pmatch/suppl_fig_2_entropy_pmatch_100_200.png", width = 6, height = 8)
ggsave("../output/figs/entropy_pmatch/suppl_fig_2_entropy_pmatch_100_200.pdf", width = 6, height = 8)

ggplot(results_entropy_all %>% filter(ymetric == "Entropy" & w_max == "w_max = 100"), aes(group = interaction(w_min,type)))+
  geom_line(aes(x = x, y = y, col = type), size=0.25)+
  geom_point(aes(x = x, y = y, fill = type, shape = w_min), size = 2, stroke = 0.25)+
  theme(axis.text.x = element_text(angle = 90, vjust = 0.5, hjust=1),
        strip.text.x = element_text(size = 11),
        strip.text.y = element_text(size = 11),
        legend.position = "bottom",
        legend.box="vertical",
        legend.title=element_blank(),
        legend.text = element_text(size = 11),
        legend.margin=margin())+
  labs(x = "Length k[s]                            Fraction Strobemers                            Length k[s]",
       y = "Entropy")+
  scale_color_manual(values = c("#F8766D", "#E76BF3", "#00B0F6", "blue", "black"))+
  scale_fill_manual(values = c("#F8766D", "#E76BF3", "#00B0F6", "blue", "black"))+
  scale_size_manual(values = c(1,1,1,1))+
  scale_shape_manual(values = c(21,24,23,22,25,4))+
  facet_grid(w_max ~ analysis, scales = "free")+
  expand_limits(x = 0, y = 0)+
  guides(shape = guide_legend(order = 2),col = guide_legend(order = 1), size = "none", linetype = "none", fill = "none")
```

```
ggsave("../output/figs/entropy_pmatch/additional_entropy_100.png", width = 7, height = 4)
ggsave("../output/figs/entropy_pmatch/additional_entropy_100.pdf", width = 7, height = 4)

ggplot(results_entropy_all %>% filter(ymetric == "p_match" & w_max == "w_max = 100"), aes(group = interaction(w_min,type)))+
  geom_line(aes(x = x, y = y, col = type), size=0.25)+
  geom_point(aes(x = x, y = y, fill = type, shape = w_min), size = 2, stroke = 0.25)+
  theme(axis.text.x = element_text(angle = 90, vjust = 0.5, hjust=1),
        strip.text.x = element_text(size = 11),
        strip.text.y = element_text(size = 11),
        legend.position = "bottom",
        legend.box="vertical",
        legend.title=element_blank(),
        legend.text = element_text(size = 11),
        legend.margin=margin())+
  labs(x = "Length k[s]                            Fraction Strobemers                            Length k[s]",
       y = "Sensitivity")+
  scale_fill_manual(values = c("#F8766D", "#E76BF3", "#00B0F6", "blue", "black"))+
  scale_color_manual(values = c("#F8766D", "#E76BF3", "#00B0F6", "blue", "black"))+
  scale_shape_manual(values = c(21,24,23,22,25,4))+
  facet_grid(w_max ~ analysis, scales = "free")+
  expand_limits(x = 0)+
  guides(shape = guide_legend(order = 2),col = guide_legend(order = 1), size = "none", linetype = "none", fill = "none")
```

```
ggsave("../output/figs/entropy_pmatch/additional_p_match_100.png", width = 7, height = 4)
ggsave("../output/figs/entropy_pmatch/additional_p_match_100.pdf", width = 7, height = 4)

ggplot(results_entropy_all %>% filter(ymetric == "Entropy" & w_max == "w_max = 200"), aes(group = interaction(w_min,type)))+
  geom_line(aes(x = x, y = y, col = type), size=0.25)+
  geom_point(aes(x = x, y = y, fill = type, shape = w_min), size = 2, stroke = 0.25)+
  theme(axis.text.x = element_text(angle = 90, vjust = 0.5, hjust=1),
        strip.text.x = element_text(size = 11),
        strip.text.y = element_text(size = 11),
        legend.position = "bottom",
        legend.box="vertical",
        legend.title=element_blank(),
        legend.text = element_text(size = 11),
        legend.margin=margin())+
  labs(x = "Length k[s]                            Fraction Strobemers                            Length k[s]",
       y = "Entropy")+
  scale_color_manual(values = c("#F8766D", "#E76BF3", "#00B0F6", "blue", "black"))+
  scale_fill_manual(values = c("#F8766D", "#E76BF3", "#00B0F6", "blue", "black"))+
  scale_size_manual(values = c(1,1,1,1))+
  scale_shape_manual(values = c(21,24,23,22,25,4))+
  facet_grid(w_max ~ analysis, scales = "free")+
  expand_limits(x = 0, y = 0)+
  guides(shape = guide_legend(order = 2),col = guide_legend(order = 1), size = "none", linetype = "none", fill = "none")
```

```
ggsave("../output/figs/entropy_pmatch/additional_entropy_200.png", width = 7, height = 4)
ggsave("../output/figs/entropy_pmatch/additional_entropy_200.pdf", width = 7, height = 4)

ggplot(results_entropy_all %>% filter(ymetric == "p_match" & w_max == "w_max = 200"), aes(group = interaction(w_min,type)))+
  geom_line(aes(x = x, y = y, col = type), size=0.25)+
  geom_point(aes(x = x, y = y, fill = type, shape = w_min), size = 2, stroke = 0.25)+
  theme(axis.text.x = element_text(angle = 90, vjust = 0.5, hjust=1),
        strip.text.x = element_text(size = 11),
        strip.text.y = element_text(size = 11),
        legend.position = "bottom",
        legend.box="vertical",
        legend.title=element_blank(),
        legend.text = element_text(size = 11),
        legend.margin=margin())+
  labs(x = "Length k[s]                            Fraction Strobemers                            Length k[s]",
       y = "Sensitivity")+
  scale_fill_manual(values = c("#F8766D", "#E76BF3", "#00B0F6", "blue", "black"))+
  scale_color_manual(values = c("#F8766D", "#E76BF3", "#00B0F6", "blue", "black"))+
  scale_shape_manual(values = c(21,24,23,22,25,4))+
  facet_grid(w_max ~ analysis, scales = "free")+
  expand_limits(x = 0)+
  guides(shape = guide_legend(order = 2),col = guide_legend(order = 1), size = "none", linetype = "none", fill = "none")
```

```
ggsave("../output/figs/entropy_pmatch/additional_p_match200.png", width = 7, height = 4)
ggsave("../output/figs/entropy_pmatch/additional_p_match200.pdf", width = 7, height = 4)
```

```
results_entropy_25_50 <- read.table("../output/data tables/p_match/10000_30_25_50_pypy.csv", header=T, sep=",") %>% 
  mutate(type = ifelse(type == "mixed-randstrobes", "mixedstrobes", type),
         type = ifelse(type == "continous altstrobes", "multistrobes", type),
         window = paste("W = [", w_min, ",", w_max, "]", sep = ""))

results_entropy_25_50 <- results_entropy_25_50 %>% mutate(x = ifelse(x == 30, 0, x))
results_entropy_25_50$type <- factor(results_entropy_25_50$type, levels= c("k-mers", "altstrobes", "randstrobes", "mixedstrobes", "multistrobes")) 


ggplot(results_entropy_25_50 %>% filter(ymetric == "Entropy"))+
  geom_point(aes(x = x, y = y, col = type, size = type))+
  geom_line(aes(x = x, y = y, col = type))+
  theme(axis.text.x = element_text(angle = 90, vjust = 0.5, hjust=1))+
  labs(x = "Length k[s]                  Fraction Strobemers                   Length k[s]",
       y = "Entropy")+
  scale_color_manual(values = c("#F8766D", "#E76BF3", "#00B0F6", "blue", "black"))+
  scale_size_manual(values = c(1,1,1,1,1))+
  facet_grid(window ~ analysis, scales = "free")+
  expand_limits(x = 0, y = 0)
```

```
ggsave("../output/figs/entropy_pmatch/additional_entropy_25_50.png", width = 9, height = 3.5)
ggsave("../output/figs/entropy_pmatch/additional_entropy_25_50.pdf", width = 9, height = 3.5)

ggplot(results_entropy_25_50 %>% filter(ymetric == "p_match"))+
  geom_point(aes(x = x, y = y, col = type))+
  theme(axis.text.x = element_text(angle = 90, vjust = 0.5, hjust=1))+
  labs(x = "Length k[s]                   Fraction Strobemers                   Length k[s]",
       y = expression(P(N[m])))+
  scale_color_manual(values = c("#F8766D", "#E76BF3", "#00B0F6", "blue", "black"))+
  facet_grid(window ~ analysis, scales = "free")+
  expand_limits(x = 0, y = 0)
```

```
ggsave("../output/figs/entropy_pmatch/additional_p_match_25_50.png", width = 9, height = 3.5)
ggsave("../output/figs/entropy_pmatch/additional_p_match_25_50.pdf", width = 9, height = 3.5)
```

```
results_entropy <- read.table("../output/data tables/p_match/fig2A_10000_30_25_50.csv", header=T, sep=",")
results_entropy$seeding <- factor(results_entropy$seeding, levels= c("k-mers", "minstrobes", "hybridstrobes", "randstrobes", "mixedstrobes", "altstrobes", "multistrobes")) 

ggplot(results_entropy)+
  geom_line(aes(x = m, y = N_m, col=seeding))+
  scale_x_continuous(breaks=c(1,5, 10, 15, 20, 25))+
  theme(legend.position = "bottom",
        legend.box="vertical",
        legend.title=element_blank(),
        legend.text = element_text(size = 10))+
  guides(col = guide_legend(nrow = 3, byrow = TRUE))+
  labs(y=expression(P(N[m] > 0)))+
  scale_color_manual(values = c("#F8766D", "#A3A500", "#00BF7D", "#00B0F6", "blue", "#E76BF3", "black"))
```

```
ggsave("../output/figs/entropy_pmatch/fig_3a_p_match-25-50.pdf", width = 4.25, height = 4)
ggsave("../output/figs/entropy_pmatch/fig_3a_p_match-25-50.png", width = 4.25, height = 4)
```

```
results_entropy_narrow_window <- rbind(
  read.table("../output/data tables/p_match/10000_30_49_50_pypy.csv", header=T, sep=","),
  read.table("../output/data tables/p_match/1000_30_99_100_pypy.csv", header=T, sep=","),
  read.table("../output/data tables/p_match/1000_30_199_200_pypy.csv", header=T, sep=",")) %>% 
  mutate(type = ifelse(type == "mixed-randstrobes", "mixedstrobes", type),
         type = ifelse(type == "continous altstrobes", "multistrobes", type),
         window = paste("W = [", w_min, ",", w_max, "]", sep = ""),
         w_max = paste("w_max = ", w_max, sep=""))

results_entropy_narrow_window$w_min <- factor(results_entropy_narrow_window$w_min, levels = c(49, 99, 199))
results_entropy_narrow_window$type <- factor(results_entropy_narrow_window$type, levels= c("k-mers", "altstrobes", "randstrobes", "mixedstrobes", "multistrobes"))
results_entropy_narrow_window$window <- factor(results_entropy_narrow_window$window, levels = c("W = [49,50]", "W = [99,100]", "W = [199,200]"))
results_entropy_narrow_window$w_max <- factor(results_entropy_narrow_window$w_max, levels = c("w_max = 50", "w_max = 100", "w_max = 200"))

results_entropy_narrow_window <- results_entropy_narrow_window %>% mutate(x = ifelse(x == 30, 0, x))

ggplot(results_entropy_narrow_window %>% mutate(ymetric = ifelse(ymetric == "p_match", "Sensitivity", ymetric)), aes(group = interaction(w_min,type)))+
  geom_line(aes(x = x, y = y, col = type), size=0.5)+
  geom_point(aes(x = x, y = y, fill = type, shape = window), size = 2, stroke = 0.25)+
  theme(axis.text.x = element_text(angle = 90, vjust = 0.5, hjust=1),
        strip.text.x = element_text(size = 11),
        strip.text.y = element_text(size = 11),
        legend.position = "bottom",
        legend.box="vertical",
        legend.title=element_blank(),
        legend.text = element_text(size = 11),
        legend.margin=margin())+
  labs(x = "Length k[s]               Fraction Strobemers             Length k[s]",
       y = "")+
  scale_fill_manual(values = c("#F8766D", "#E76BF3", "#00B0F6", "blue", "black"))+
  scale_color_manual(values = c("#F8766D", "#E76BF3", "#00B0F6", "blue", "black"))+
  scale_shape_manual(values = c(21,24,23,22,25))+
  facet_nested(w_max+ymetric ~ analysis, scales = "free")+
  expand_limits(x = 0)+
  guides(shape = guide_legend(order = 2),col = guide_legend(order = 1), size = "none", linetype = "none", fill = "none")
```

```
ggsave("../output/figs/entropy_pmatch/suppl_fig_3_entropy_pmatch_small_window.png", width = 6, height = 10)
ggsave("../output/figs/entropy_pmatch/suppl_fig_3_entropy_pmatch_small_window.pdf", width = 6, height = 10)
```

## Seed Uniqueness (E-hits)

```
results_esize <- read.table(file = "../output/data tables/esize", header = TRUE, sep = ",") %>% mutate(datastructure = ifelse(datastructure == "mixedrandstrobes", "mixedstrobes", datastructure))
results_esize$datastructure = factor(results_esize$datastructure, levels=c('kmers', 'randstrobes', 'mixedstrobes', 'altstrobes', 'multistrobes'))

ggplot(results_esize)+
  geom_line(aes(x = k, y = ehits, col = datastructure, linetype = datastructure))+
  geom_point(aes(x = k, y = ehits, col = datastructure, shape = datastructure))+
  scale_linetype_manual(values=c("solid", "longdash", "longdash", "longdash", "longdash"))+
  scale_shape_manual(values = c(16,24,24,25,19))+
  scale_color_manual(values = c("#F8766D", "#00B0F6", "blue", "#E76BF3", "black"))+
  theme(strip.text.x = element_text(size = 11),
        strip.text.y = element_text(size = 11),
        legend.position = "bottom",
        legend.box="vertical",
        legend.title=element_blank(),
        legend.text = element_text(size = 11))+
  guides(linetype = guide_legend(nrow = 2, byrow = TRUE))+
  ylab("E-hits")+
  scale_x_continuous(limits = c(18, 36))+
  scale_y_log10(breaks = c(10,20,30,40,50))
```

```
ggsave("../output/figs/uniqueness_esize/fig_3b_ehits.png", width = 4.25, height = 4)
ggsave("../output/figs/uniqueness_esize/fig_3b_ehits.pdf", width = 4.25, height = 4)
```

## Sequence Matching Results (E.coli ONT Reads)

```
kmers <- read.table("../output/data tables/matching_analysis_bio_collinear/kmers (Summary).txt", sep="&") %>% mutate(method = "kmers", strobe_fraction = 0)

mixedrandstrobes10 <- read.table("../output/data tables/matching_analysis_bio_collinear/mixedrandstrobe10 (Summary).txt", sep="&") %>% mutate(method = "randstrobes", strobe_fraction = 10)
mixedrandstrobes20 <- read.table("../output/data tables/matching_analysis_bio_collinear/mixedrandstrobe20 (Summary).txt", sep="&") %>% mutate(method = "randstrobes", strobe_fraction = 20)
mixedrandstrobes30 <- read.table("../output/data tables/matching_analysis_bio_collinear/mixedrandstrobe30 (Summary).txt", sep="&") %>% mutate(method = "randstrobes", strobe_fraction = 30)
mixedrandstrobes40 <- read.table("../output/data tables/matching_analysis_bio_collinear/mixedrandstrobe40 (Summary).txt", sep="&") %>% mutate(method = "randstrobes", strobe_fraction = 40)
mixedrandstrobes50 <- read.table("../output/data tables/matching_analysis_bio_collinear/mixedrandstrobe50 (Summary).txt", sep="&") %>% mutate(method = "randstrobes", strobe_fraction = 50)
mixedrandstrobes60 <- read.table("../output/data tables/matching_analysis_bio_collinear/mixedrandstrobe60 (Summary).txt", sep="&") %>% mutate(method = "randstrobes", strobe_fraction = 60)
mixedrandstrobes70 <- read.table("../output/data tables/matching_analysis_bio_collinear/mixedrandstrobe70 (Summary).txt", sep="&") %>% mutate(method = "randstrobes", strobe_fraction = 70)
mixedrandstrobes80 <- read.table("../output/data tables/matching_analysis_bio_collinear/mixedrandstrobe80 (Summary).txt", sep="&") %>% mutate(method = "randstrobes", strobe_fraction = 80)
mixedrandstrobes90 <- read.table("../output/data tables/matching_analysis_bio_collinear/mixedrandstrobe90 (Summary).txt", sep="&") %>% mutate(method = "randstrobes", strobe_fraction = 90)
randstrobes <- read.table("../output/data tables/matching_analysis_bio_collinear/randstrobe (Summary).txt", sep="&") %>% mutate(method = "randstrobes", strobe_fraction = 100)

mixedhybridstrobes10 <- read.table("../output/data tables/matching_analysis_bio_collinear/mixedhybridstrobe10 (Summary).txt", sep="&") %>% mutate(method = "hybridstrobes", strobe_fraction = 10)
mixedhybridstrobes20 <- read.table("../output/data tables/matching_analysis_bio_collinear/mixedhybridstrobe20 (Summary).txt", sep="&") %>% mutate(method = "hybridstrobes", strobe_fraction = 20)
mixedhybridstrobes30 <- read.table("../output/data tables/matching_analysis_bio_collinear/mixedhybridstrobe30 (Summary).txt", sep="&") %>% mutate(method = "hybridstrobes", strobe_fraction = 30)
mixedhybridstrobes40 <- read.table("../output/data tables/matching_analysis_bio_collinear/mixedhybridstrobe40 (Summary).txt", sep="&") %>% mutate(method = "hybridstrobes", strobe_fraction = 40)
mixedhybridstrobes50 <- read.table("../output/data tables/matching_analysis_bio_collinear/mixedhybridstrobe50 (Summary).txt", sep="&") %>% mutate(method = "hybridstrobes", strobe_fraction = 50)
mixedhybridstrobes60 <- read.table("../output/data tables/matching_analysis_bio_collinear/mixedhybridstrobe60 (Summary).txt", sep="&") %>% mutate(method = "hybridstrobes", strobe_fraction = 60)
mixedhybridstrobes70 <- read.table("../output/data tables/matching_analysis_bio_collinear/mixedhybridstrobe70 (Summary).txt", sep="&") %>% mutate(method = "hybridstrobes", strobe_fraction = 70)
mixedhybridstrobes80 <- read.table("../output/data tables/matching_analysis_bio_collinear/mixedhybridstrobe80 (Summary).txt", sep="&") %>% mutate(method = "hybridstrobes", strobe_fraction = 80)
mixedhybridstrobes90 <- read.table("../output/data tables/matching_analysis_bio_collinear/mixedhybridstrobe90 (Summary).txt", sep="&") %>% mutate(method = "hybridstrobes", strobe_fraction = 90)
hybridstrobes <- read.table("../output/data tables/matching_analysis_bio_collinear/hybridstrobe (Summary).txt", sep="&") %>% mutate(method = "hybridstrobes", strobe_fraction = 100)

mixedminstrobes10 <- read.table("../output/data tables/matching_analysis_bio_collinear/mixedminstrobe10 (Summary).txt", sep="&") %>% mutate(method = "minstrobes", strobe_fraction = 10)
mixedminstrobes20 <- read.table("../output/data tables/matching_analysis_bio_collinear/mixedminstrobe20 (Summary).txt", sep="&") %>% mutate(method = "minstrobes", strobe_fraction = 20)
mixedminstrobes30 <- read.table("../output/data tables/matching_analysis_bio_collinear/mixedminstrobe30 (Summary).txt", sep="&") %>% mutate(method = "minstrobes", strobe_fraction = 30)
mixedminstrobes40 <- read.table("../output/data tables/matching_analysis_bio_collinear/mixedminstrobe40 (Summary).txt", sep="&") %>% mutate(method = "minstrobes", strobe_fraction = 40)
mixedminstrobes50 <- read.table("../output/data tables/matching_analysis_bio_collinear/mixedminstrobe50 (Summary).txt", sep="&") %>% mutate(method = "minstrobes", strobe_fraction = 50)
mixedminstrobes60 <- read.table("../output/data tables/matching_analysis_bio_collinear/mixedminstrobe60 (Summary).txt", sep="&") %>% mutate(method = "minstrobes", strobe_fraction = 60)
mixedminstrobes70 <- read.table("../output/data tables/matching_analysis_bio_collinear/mixedminstrobe70 (Summary).txt", sep="&") %>% mutate(method = "minstrobes", strobe_fraction = 70)
mixedminstrobes80 <- read.table("../output/data tables/matching_analysis_bio_collinear/mixedminstrobe80 (Summary).txt", sep="&") %>% mutate(method = "minstrobes", strobe_fraction = 80)
mixedminstrobes90 <- read.table("../output/data tables/matching_analysis_bio_collinear/mixedminstrobe90 (Summary).txt", sep="&") %>% mutate(method = "minstrobes", strobe_fraction = 90)
minstrobes <- read.table("../output/data tables/matching_analysis_bio_collinear/minstrobe (Summary).txt", sep="&") %>% mutate(method = "minstrobes", strobe_fraction = 100) #check

mixedaltstrobes10 <- read.table("../output/data tables/matching_analysis_bio_collinear/mixedaltstrobe10 (Summary).txt", sep="&") %>% mutate(method = "altstrobes", strobe_fraction = 10)
mixedaltstrobes20 <- read.table("../output/data tables/matching_analysis_bio_collinear/mixedaltstrobe20 (Summary).txt", sep="&") %>% mutate(method = "altstrobes", strobe_fraction = 20)
mixedaltstrobes30 <- read.table("../output/data tables/matching_analysis_bio_collinear/mixedaltstrobe30 (Summary).txt", sep="&") %>% mutate(method = "altstrobes", strobe_fraction = 30)
mixedaltstrobes40 <- read.table("../output/data tables/matching_analysis_bio_collinear/mixedaltstrobe40 (Summary).txt", sep="&") %>% mutate(method = "altstrobes", strobe_fraction = 40)
mixedaltstrobes50 <- read.table("../output/data tables/matching_analysis_bio_collinear/mixedaltstrobe50 (Summary).txt", sep="&") %>% mutate(method = "altstrobes", strobe_fraction = 50)
mixedaltstrobes60 <- read.table("../output/data tables/matching_analysis_bio_collinear/mixedaltstrobe60 (Summary).txt", sep="&") %>% mutate(method = "altstrobes", strobe_fraction = 60)
mixedaltstrobes70 <- read.table("../output/data tables/matching_analysis_bio_collinear/mixedaltstrobe70 (Summary).txt", sep="&") %>% mutate(method = "altstrobes", strobe_fraction = 70)
mixedaltstrobes80 <- read.table("../output/data tables/matching_analysis_bio_collinear/mixedaltstrobe80 (Summary).txt", sep="&") %>% mutate(method = "altstrobes", strobe_fraction = 80)
mixedaltstrobes90 <- read.table("../output/data tables/matching_analysis_bio_collinear/mixedaltstrobe90 (Summary).txt", sep="&") %>% mutate(method = "altstrobes", strobe_fraction = 90)
altstrobes <- read.table("../output/data tables/matching_analysis_bio_collinear/altstrobe (Summary).txt", sep="&") %>% mutate(method = "altstrobes", strobe_fraction = 100)
multistrobes <- read.table("../output/data tables/matching_analysis_bio_collinear/multistrobe (Summary).txt", sep="&") %>% mutate(method = "multistrobes", strobe_fraction = 100)

results_coll <- rbind(
  kmers, 
  mixedhybridstrobes10, mixedhybridstrobes20, mixedhybridstrobes30, mixedhybridstrobes40, mixedhybridstrobes50, 
  mixedhybridstrobes60, mixedhybridstrobes70, mixedhybridstrobes80, mixedhybridstrobes90, hybridstrobes,
  mixedminstrobes10, mixedminstrobes20, mixedminstrobes30, mixedminstrobes40, mixedminstrobes50, 
  mixedminstrobes60, mixedminstrobes70, mixedminstrobes80, mixedminstrobes90, minstrobes,
  mixedrandstrobes10, mixedrandstrobes20, mixedrandstrobes30, mixedrandstrobes40, mixedrandstrobes50, 
  mixedrandstrobes60, mixedrandstrobes70, mixedrandstrobes80, mixedrandstrobes90, randstrobes,
  mixedaltstrobes10, mixedaltstrobes20, mixedaltstrobes30, mixedaltstrobes40, #mixedaltstrobes50, 
  mixedaltstrobes60, mixedaltstrobes70, mixedaltstrobes80, mixedaltstrobes90, altstrobes, 
  multistrobes
  ) %>% rename("query" = "V1", "length" = "V2", "mp" = "V3", "sc" = "V4", "mc" = "V5", "esize" = "V6", "coll_esize" = "V7", "m" = "V8", "nm" = "V9", "ANI" = "V10")
results_coll$method <- factor(results_coll$method, levels = c("kmers", "minstrobes", "hybridstrobes", "randstrobes", "altstrobes", "multistrobes"))

results_coll_rand_alt <- rbind(mixedrandstrobes80 %>% mutate(method = "mixedrandstrobes80"), randstrobes, altstrobes, multistrobes)%>% rename("query" = "V1", "length" = "V2", "mp" = "V3", "sc" = "V4", "mc" = "V5", "esize" = "V6", "coll_esize" = "V7", "m" = "V8", "nm" = "V9", "ANI" = "V10")
results_coll_rand_alt$method <- factor(results_coll_rand_alt$method, levels =c("mixedrandstrobes80", "randstrobes", "altstrobes", "multistrobes"))

n_protocol <- results_coll %>% group_by(method, strobe_fraction) %>% summarise(tot_length = sum(length))
```

```
## `summarise()` has grouped output by 'method'. You can override using the
## `.groups` argument.
```

```
results_coll_summarized <- results_coll %>% 
  mutate(mp = mp * length, sc = sc * length, mc = mc * length, esize = esize * length) %>% 
  group_by(method, strobe_fraction) %>% summarise(length = mean(length), mp = sum(mp), sc = sum(sc), mc = sum(mc), esize = sum(esize)) %>% 
  left_join(n_protocol, by=c("method", "strobe_fraction")) %>% 
  mutate(mp = mp/tot_length, sc = sc/tot_length, mc = mc/tot_length, esize = esize/tot_length)
```

```
## `summarise()` has grouped output by 'method'. You can override using the
## `.groups` argument.
```

```
results_coll_summarized$method <- factor(results_coll_summarized$method, levels = c("kmers", "minstrobes", "hybridstrobes", "randstrobes", "altstrobes", "multistrobes"))

results_coll_comparison <- left_join(randstrobes, mixedrandstrobes80, by = c("V1", "V2")) %>% 
  left_join(altstrobes, by = c("V1", "V2")) %>% left_join(multistrobes, by = c("V1", "V2")) %>% 
  mutate(mp_mixed = V3.y/V3.x, mp_alt = V3.x.x/V3.x, mp_mult = V3.y.y/V3.x,
         sc_mixed = V4.y/V4.x, sc_alt = V4.x.x/V4.x, sc_mult = V4.y.y/V4.x,
         mc_mixed = V5.y/V5.x, mc_alt = V5.x.x/V5.x, mc_mult = V5.y.y/V5.x,
         esize_mixed = V6.y/V6.x, esize_alt = V6.x.x/V6.x, esize_mult = V6.y.y/V6.x) %>% 
  dplyr::select("length"=V2,mp_mixed, mp_alt, mp_mult, sc_mixed, sc_alt, sc_mult, mc_mixed, mc_alt, mc_mult, esize_mixed, esize_alt, esize_mult)
```

```
p <- ggplot(results_coll_summarized)+
  geom_point(aes(x = strobe_fraction, y = mp, col = method, shape = method))+
  geom_line(aes(x = strobe_fraction, y = mp, col = method))+
  theme(axis.text.x = element_text(angle = 90, vjust = 0.5, hjust=1),
        legend.position = "none")+
  scale_shape_manual(values = c(16,22,23,24,25,21))+
  scale_color_manual(values = c("#F8766D", "#A3A500", "#00BF7D", "#00B0F6", "#E76BF3", "black"))+
  labs(x = "Strobe Fraction [%]",
       y = "Matches [%]")
ggsave("../output/figs/matching_analysis_bio/fig_4a_matches_(2,15,25,50).png", height = 3, width = 3)
ggsave("../output/figs/matching_analysis_bio/fig_4a_matches_(2,15,25,50).pdf", height = 3, width = 3)

p <- ggplot(results_coll_summarized)+
  geom_point(aes(x = strobe_fraction, y = sc, col = method, shape = method))+
  geom_line(aes(x = strobe_fraction, y = sc, col = method))+
  theme(axis.text.x = element_text(angle = 90, vjust = 0.5, hjust=1),
        legend.position = "none")+
  scale_shape_manual(values = c(16,22,23,24,25,21))+
  scale_color_manual(values = c("#F8766D", "#A3A500", "#00BF7D", "#00B0F6", "#E76BF3", "black"))+
  labs(x = "Strobe Fraction [%]",
       y = "Sequence Coverage [%]")
ggsave("../output/figs/matching_analysis_bio/fig_4c_sequence_coverage_(2,15,25,50).png", height = 3, width = 3)
ggsave("../output/figs/matching_analysis_bio/fig_4c_sequence_coverage_(2,15,25,50).pdf", height = 3, width = 3)

p <- ggplot(results_coll_summarized)+
  geom_point(aes(x = strobe_fraction, y = mc, col = method, shape = method))+
  geom_line(aes(x = strobe_fraction, y = mc, col = method))+
  theme(axis.text.x = element_text(angle = 90, vjust = 0.5, hjust=1),
        legend.position = "none")+
  scale_shape_manual(values = c(16,22,23,24,25,21))+
  scale_color_manual(values = c("#F8766D", "#A3A500", "#00BF7D", "#00B0F6", "#E76BF3", "black"))+
  labs(x = "Strobe Fraction [%]",
       y = "Match Coverage [%]")
ggsave("../output/figs/matching_analysis_bio/fig_4b_match_coverage_(2,15,25,50).png", height = 3, width = 3)
ggsave("../output/figs/matching_analysis_bio/fig_4b_match_coverage_(2,15,25,50).pdf", height = 3, width = 3)

p <- ggplot(results_coll_summarized)+
  geom_point(aes(x = strobe_fraction, y = esize, col = method, shape = method))+
  geom_line(aes(x = strobe_fraction, y = esize, col = method))+
  theme(axis.text.x = element_text(angle = 90, vjust = 0.5, hjust=1),
        legend.position = "none")+
  scale_shape_manual(values = c(16,22,23,24,25,21))+
  scale_color_manual(values = c("#F8766D", "#A3A500", "#00BF7D", "#00B0F6", "#E76BF3", "black"))+
  labs(x = "Strobe Fraction [%]",
       y = "Expected Island Size")
ggsave("../output/figs/matching_analysis_bio/fig_3d_expected_island_size_(2,15,25,50).png", height = 3, width = 3)
ggsave("../output/figs/matching_analysis_bio/fig_4d_expected_island_size_(2,15,25,50).pdf", height = 3, width = 3)
```

```
ggplot(results_coll %>% filter(strobe_fraction == 0 | strobe_fraction == 100), aes(x = length, y = mp, col = method))+
  geom_point(size= 0.2, alpha = 0.2)+
  geom_smooth(size = 0.6, se = F)+
  theme(axis.text.x = element_text(angle = 90, vjust = 0.5, hjust=1),
        legend.position = "none")+
  scale_color_manual(values = c("#F8766D", "#A3A500", "#00BF7D", "#00B0F6", "#E76BF3", "black"))+
  labs(x = "Length [bp]",
       y = "Matches [%]")+
  ylim(0, 25)
```

```
## `geom_smooth()` using method = 'gam' and formula = 'y ~ s(x, bs = "cs")'
```

```
## Warning: Removed 1 rows containing non-finite values (`stat_smooth()`).
```

```
## Warning: Removed 1 rows containing missing values (`geom_point()`).
```

```
ggsave("../output/figs/matching_analysis_bio/suppl_fig_S7_matches_(2,15,25,50).png", height = 3, width = 3)
```

```
## `geom_smooth()` using method = 'gam' and formula = 'y ~ s(x, bs = "cs")'
```

```
## Warning: Removed 1 rows containing non-finite values (`stat_smooth()`).
## Removed 1 rows containing missing values (`geom_point()`).
```

```
ggsave("../output/figs/matching_analysis_bio/suppl_fig_S7_matches_(2,15,25,50).pdf", height = 3, width = 3)
```

```
## `geom_smooth()` using method = 'gam' and formula = 'y ~ s(x, bs = "cs")'
```

```
## Warning: Removed 1 rows containing non-finite values (`stat_smooth()`).
## Removed 1 rows containing missing values (`geom_point()`).
```

```
ggplot(results_coll %>% filter(strobe_fraction == 0 | strobe_fraction == 100), aes(x = length, y = sc, col = method))+
  geom_point(size= 0.2, alpha = 0.2)+
  geom_smooth(size = 0.6, se = F)+
  theme(axis.text.x = element_text(angle = 90, vjust = 0.5, hjust=1),
        legend.position = "none")+
  scale_color_manual(values = c("#F8766D", "#A3A500", "#00BF7D", "#00B0F6", "#E76BF3", "black"))+
  labs(x = "Length [bp]",
       y = "Sequence Coverage [%]")+
  ylim(0, 100)
```

```
## `geom_smooth()` using method = 'gam' and formula = 'y ~ s(x, bs = "cs")'
```

```
ggsave("../output/figs/matching_analysis_bio/suppl_fig_S7_sequence_coverage_(2,15,25,50).png", height = 3, width = 3)
```

```
## `geom_smooth()` using method = 'gam' and formula = 'y ~ s(x, bs = "cs")'
```

```
ggsave("../output/figs/matching_analysis_bio/suppl_fig_S7_sequence_coverage_(2,15,25,50).pdf", height = 3, width = 3)
```

```
## `geom_smooth()` using method = 'gam' and formula = 'y ~ s(x, bs = "cs")'
```

```
ggplot(results_coll %>% filter(strobe_fraction == 0 | strobe_fraction == 100), aes(x = length, y = mc, col = method))+
  geom_point(size= 0.2, alpha = 0.2)+
  geom_smooth(size = 0.6, se = F)+
  theme(axis.text.x = element_text(angle = 90, vjust = 0.5, hjust=1),
        legend.position = "none")+
  scale_color_manual(values = c("#F8766D", "#A3A500", "#00BF7D", "#00B0F6", "#E76BF3", "black"))+
  labs(x = "Length [bp]",
       y = "Match Coverage [%]")+
  ylim(0, 100)
```

```
## `geom_smooth()` using method = 'gam' and formula = 'y ~ s(x, bs = "cs")'
```

```
ggsave("../output/figs/matching_analysis_bio/suppl_fig_S7_match_coverage_(2,15,25,50).png", height = 3, width = 3)
```

```
## `geom_smooth()` using method = 'gam' and formula = 'y ~ s(x, bs = "cs")'
```

```
ggsave("../output/figs/matching_analysis_bio/suppl_fig_S7_match_coverage_(2,15,25,50).pdf", height = 3, width = 3)
```

```
## `geom_smooth()` using method = 'gam' and formula = 'y ~ s(x, bs = "cs")'
```

```
ggplot(results_coll %>% filter(strobe_fraction == 0 | strobe_fraction == 100), aes(x = length, y = esize, col = method))+
  geom_point(size= 0.2, alpha = 0.2)+
  geom_smooth(size = 0.6, se = F)+
  theme(axis.text.x = element_text(angle = 90, vjust = 0.5, hjust=1),
        legend.position = "none")+
  scale_color_manual(values = c("#F8766D", "#A3A500", "#00BF7D", "#00B0F6", "#E76BF3", "black"))+
  labs(x = "Length [bp]",
       y = "Expected Island Size [bp]")+
  ylim(0, 1200)
```

```
## `geom_smooth()` using method = 'gam' and formula = 'y ~ s(x, bs = "cs")'
```

```
ggsave("../output/figs/matching_analysis_bio/suppl_fig_S7_expected_island_size_(2,15,25,50).png", height = 3, width = 3)
```

```
## `geom_smooth()` using method = 'gam' and formula = 'y ~ s(x, bs = "cs")'
```

```
ggsave("../output/figs/matching_analysis_bio/suppl_fig_S7_expected_island_size_(2,15,25,50).pdf", height = 3, width = 3)
```

```
## `geom_smooth()` using method = 'gam' and formula = 'y ~ s(x, bs = "cs")'
```

```
ggplot(results_coll_comparison)+
  geom_point(aes(x = length, y = mp_mixed), col = "blue", size= 0.2, alpha = 0.2)+
  geom_smooth(aes(x = length, y = mp_mixed), col = "blue", size = 0.4, se = F)+
  geom_point(aes(x = length, y = mp_alt), col = "#E76BF3", size= 0.2, alpha = 0.2)+
  geom_smooth(aes(x = length, y = mp_alt), col = "#E76BF3", size = 0.4, se = F)+
  geom_point(aes(x = length, y = mp_mult), col = "black", size= 0.2, alpha = 0.2)+
  geom_smooth(aes(x = length, y = mp_mult), col = "black", size = 0.4, se = F)+
  geom_line(aes(x=length, y = 1), col = "#00B0F6")+
  theme(axis.text.x = element_text(angle = 90, vjust = 0.5, hjust=1), 
        legend.position = "none")+
  #scale_y_continuous(trans = log2_trans(), breaks = c(0.9, 1.0, 1.1, 1.2, 1.3, 1.4))+
  labs(x = "Length [bp]",
       y = "Matches [normalized]")
```

```
## `geom_smooth()` using method = 'gam' and formula = 'y ~ s(x, bs = "cs")'
## `geom_smooth()` using method = 'gam' and formula = 'y ~ s(x, bs = "cs")'
## `geom_smooth()` using method = 'gam' and formula = 'y ~ s(x, bs = "cs")'
```

```
ggsave("../output/figs/matching_analysis_bio/suppl_fig_S8_matches_detail_normalized_(2,15,25,50).png", height = 3, width = 3)
```

```
## `geom_smooth()` using method = 'gam' and formula = 'y ~ s(x, bs = "cs")'
## `geom_smooth()` using method = 'gam' and formula = 'y ~ s(x, bs = "cs")'
## `geom_smooth()` using method = 'gam' and formula = 'y ~ s(x, bs = "cs")'
```

```
ggsave("../output/figs/matching_analysis_bio/suppl_fig_S8_matches_detail_normalized_(2,15,25,50).pdf", height = 3, width = 3)
```

```
## `geom_smooth()` using method = 'gam' and formula = 'y ~ s(x, bs = "cs")'
## `geom_smooth()` using method = 'gam' and formula = 'y ~ s(x, bs = "cs")'
## `geom_smooth()` using method = 'gam' and formula = 'y ~ s(x, bs = "cs")'
```

```
ggplot(results_coll_comparison)+
  geom_point(aes(x = length, y = sc_mixed), col = "blue", size= 0.2, alpha = 0.2)+
  geom_smooth(aes(x = length, y = sc_mixed), col = "blue", size = 0.4, se = F)+
  geom_point(aes(x = length, y = sc_alt), col = "#E76BF3", size= 0.2, alpha = 0.2)+
  geom_smooth(aes(x = length, y = sc_alt), col = "#E76BF3", size = 0.4, se = F)+
  geom_point(aes(x = length, y = sc_mult), col = "black", size= 0.2, alpha = 0.2)+
  geom_smooth(aes(x = length, y = sc_mult), col = "black", size = 0.4, se = F)+
  geom_line(aes(x=length, y = 1), col = "#00B0F6")+
  theme(axis.text.x = element_text(angle = 90, vjust = 0.5, hjust=1), 
        legend.position = "none")+
  #scale_y_continuous(trans = log2_trans(), breaks = c(0.9, 1.0, 1.1, 1.2, 1.3, 1.4))+
  labs(x = "Length [bp]",
       y = "Sequence Coverage [normalized]")
```

```
## `geom_smooth()` using method = 'gam' and formula = 'y ~ s(x, bs = "cs")'
## `geom_smooth()` using method = 'gam' and formula = 'y ~ s(x, bs = "cs")'
## `geom_smooth()` using method = 'gam' and formula = 'y ~ s(x, bs = "cs")'
```

```
ggsave("../output/figs/matching_analysis_bio/suppl_fig_S8_sequence_coverage_detail_normalized_(2,15,25,50).png", height = 3, width = 3)
```

```
## `geom_smooth()` using method = 'gam' and formula = 'y ~ s(x, bs = "cs")'
## `geom_smooth()` using method = 'gam' and formula = 'y ~ s(x, bs = "cs")'
## `geom_smooth()` using method = 'gam' and formula = 'y ~ s(x, bs = "cs")'
```

```
ggsave("../output/figs/matching_analysis_bio/suppl_fig_S8_sequence_coverage_detail_normalized_(2,15,25,50).pdf", height = 3, width = 3)
```

```
## `geom_smooth()` using method = 'gam' and formula = 'y ~ s(x, bs = "cs")'
## `geom_smooth()` using method = 'gam' and formula = 'y ~ s(x, bs = "cs")'
## `geom_smooth()` using method = 'gam' and formula = 'y ~ s(x, bs = "cs")'
```

```
ggplot(results_coll_comparison)+
  geom_point(aes(x = length, y = mc_mixed), col = "blue", size= 0.2, alpha = 0.2)+
  geom_smooth(aes(x = length, y = mc_mixed), col = "blue", size = 0.4, se = F)+
  geom_point(aes(x = length, y = mc_alt), col = "#E76BF3", size= 0.2, alpha = 0.2)+
  geom_smooth(aes(x = length, y = mc_alt), col = "#E76BF3", size = 0.4, se = F)+
  geom_point(aes(x = length, y = mc_mult), col = "black", size= 0.2, alpha = 0.2)+
  geom_smooth(aes(x = length, y = mc_mult), col = "black", size = 0.4, se = F)+
  geom_line(aes(x=length, y = 1), col = "#00B0F6")+
  theme(axis.text.x = element_text(angle = 90, vjust = 0.5, hjust=1), 
        legend.position = "none")+
  #scale_y_continuous(trans = log2_trans(), breaks = c(0.8, 0.9, 1.0, 1.1, 1.2))+
  labs(x = "Length [bp]",
       y = "Match Coverage [normalized]")
```

```
## `geom_smooth()` using method = 'gam' and formula = 'y ~ s(x, bs = "cs")'
## `geom_smooth()` using method = 'gam' and formula = 'y ~ s(x, bs = "cs")'
## `geom_smooth()` using method = 'gam' and formula = 'y ~ s(x, bs = "cs")'
```

```
ggsave("../output/figs/matching_analysis_bio/suppl_fig_S8_match_coverage_detail_normalized_(2,15,25,50).png", height = 3, width = 3)
```

```
## `geom_smooth()` using method = 'gam' and formula = 'y ~ s(x, bs = "cs")'
## `geom_smooth()` using method = 'gam' and formula = 'y ~ s(x, bs = "cs")'
## `geom_smooth()` using method = 'gam' and formula = 'y ~ s(x, bs = "cs")'
```

```
ggsave("../output/figs/matching_analysis_bio/suppl_fig_S8_match_coverage_detail_normalized_(2,15,25,50).pdf", height = 3, width = 3)
```

```
## `geom_smooth()` using method = 'gam' and formula = 'y ~ s(x, bs = "cs")'
## `geom_smooth()` using method = 'gam' and formula = 'y ~ s(x, bs = "cs")'
## `geom_smooth()` using method = 'gam' and formula = 'y ~ s(x, bs = "cs")'
```

```
ggplot(results_coll_comparison)+
  geom_point(aes(x = length, y = esize_mixed), col = "blue", size= 0.2, alpha = 0.2)+
  geom_smooth(aes(x = length, y = esize_mixed), col = "blue", size = 0.4, se = F)+
  geom_point(aes(x = length, y = esize_alt), col = "#E76BF3", size= 0.2, alpha = 0.2)+
  geom_smooth(aes(x = length, y = esize_alt), col = "#E76BF3", size = 0.4, se = F)+
  geom_point(aes(x = length, y = esize_mult), col = "black", size= 0.2, alpha = 0.2)+
  geom_smooth(aes(x = length, y = esize_mult), col = "black", size = 0.4, se = F)+
  geom_line(aes(x=length, y = 1), col = "#00B0F6")+
  theme(axis.text.x = element_text(angle = 90, vjust = 0.5, hjust=1), 
        legend.position = "none")+
  #scale_y_continuous(trans = log2_trans(), breaks = c(0.5, 0.75, 1.0, 1.25, 1.5, 1.75, 2))+
  labs(x = "Length [bp]",
       y = "Expected Island Size [normalized]")
```

```
## `geom_smooth()` using method = 'gam' and formula = 'y ~ s(x, bs = "cs")'
## `geom_smooth()` using method = 'gam' and formula = 'y ~ s(x, bs = "cs")'
## `geom_smooth()` using method = 'gam' and formula = 'y ~ s(x, bs = "cs")'
```

```
ggsave("../output/figs/matching_analysis_bio/suppl_fig_S8_expected_island_size_detail_normalized_(2,15,25,50).png", height = 3, width = 3)
```

```
## `geom_smooth()` using method = 'gam' and formula = 'y ~ s(x, bs = "cs")'
## `geom_smooth()` using method = 'gam' and formula = 'y ~ s(x, bs = "cs")'
## `geom_smooth()` using method = 'gam' and formula = 'y ~ s(x, bs = "cs")'
```

```
ggsave("../output/figs/matching_analysis_bio/suppl_fig_S8_expected_island_size_detail_normalized_(2,15,25,50).pdf", height = 3, width = 3)
```

```
## `geom_smooth()` using method = 'gam' and formula = 'y ~ s(x, bs = "cs")'
## `geom_smooth()` using method = 'gam' and formula = 'y ~ s(x, bs = "cs")'
## `geom_smooth()` using method = 'gam' and formula = 'y ~ s(x, bs = "cs")'
```

```
ggplot(results_coll_rand_alt, aes(x = length, y = mp, col = method))+
  geom_point(size= 0.2, alpha = 0.2)+
  geom_smooth(size = 0.4, se = F)+
  theme(axis.text.x = element_text(angle = 90, vjust = 0.5, hjust=1),
        legend.position = "none")+
  scale_color_manual(values = c("blue", "#00B0F6", "#E76BF3", "black"))+
  scale_linetype_manual(values = c("dotted", "solid", "solid", "solid"))+ 
  labs(x = "Length [bp]",
       y = "Matches [%]")
```

```
## `geom_smooth()` using method = 'gam' and formula = 'y ~ s(x, bs = "cs")'
```

```
ggsave("../output/figs/matching_analysis_bio/additional_matches_detail_(2,15,25,50).png", height = 3, width = 3)
```

```
## `geom_smooth()` using method = 'gam' and formula = 'y ~ s(x, bs = "cs")'
```

```
ggsave("../output/figs/matching_analysis_bio/additional_matches_detail_(2,15,25,50).pdf", height = 3, width = 3)
```

```
## `geom_smooth()` using method = 'gam' and formula = 'y ~ s(x, bs = "cs")'
```

```
ggplot(results_coll_rand_alt, aes(x = length, y = sc, col = method))+
  geom_point(size= 0.2, alpha = 0.2)+
  geom_smooth(size = 0.4, se = F)+
  theme(axis.text.x = element_text(angle = 90, vjust = 0.5, hjust=1),
        legend.position = "none")+
  scale_color_manual(values = c("blue", "#00B0F6", "#E76BF3", "black"))+
  scale_linetype_manual(values = c("dotted", "solid", "solid", "solid"))+  
  labs(x = "Length [bp]",
       y = "Sequence Coverage [%]")
```

```
## `geom_smooth()` using method = 'gam' and formula = 'y ~ s(x, bs = "cs")'
```

```
ggsave("../output/figs/matching_analysis_bio/additional_sequence_coverage_detail_(2,15,25,50).png", height = 3, width = 3)
```

```
## `geom_smooth()` using method = 'gam' and formula = 'y ~ s(x, bs = "cs")'
```

```
ggsave("../output/figs/matching_analysis_bio/additional_sequence_coverage_detail_(2,15,25,50).pdf", height = 3, width = 3)
```

```
## `geom_smooth()` using method = 'gam' and formula = 'y ~ s(x, bs = "cs")'
```

```
ggplot(results_coll_rand_alt, aes(x = length, y = mc, col = method))+
  geom_point(size= 0.2, alpha = 0.2)+
  geom_smooth(size = 0.4, se = F)+
  theme(axis.text.x = element_text(angle = 90, vjust = 0.5, hjust=1),
        legend.position = "none")+
  scale_color_manual(values = c("blue", "#00B0F6", "#E76BF3", "black"))+
  scale_linetype_manual(values = c("dotted", "solid", "solid", "solid"))+  
  labs(x = "Length [bp]",
       y = "Match Coverage [%]")
```

```
## `geom_smooth()` using method = 'gam' and formula = 'y ~ s(x, bs = "cs")'
```

```
ggsave("../output/figs/matching_analysis_bio/additional_match_coverage_detail_(2,15,25,50).png", height = 3, width = 3)
```

```
## `geom_smooth()` using method = 'gam' and formula = 'y ~ s(x, bs = "cs")'
```

```
ggsave("../output/figs/matching_analysis_bio/additional_match_coverage_detail_(2,15,25,50).pdf", height = 3, width = 3)
```

```
## `geom_smooth()` using method = 'gam' and formula = 'y ~ s(x, bs = "cs")'
```

```
ggplot(results_coll_rand_alt, aes(x = length, y = esize, col = method))+
  geom_point(size= 0.2, alpha = 0.2)+
  geom_smooth(size = 0.4, se = F)+
  theme(axis.text.x = element_text(angle = 90, vjust = 0.5, hjust=1),
        legend.position = "none")+
  scale_color_manual(values = c("blue", "#00B0F6", "#E76BF3", "black"))+
  scale_linetype_manual(values = c("dotted", "solid", "solid", "solid"))+ 
  scale_y_log10()+
  labs(x = "Length [bp]",
       y = "Expected Island Size [bp]")
```

```
## `geom_smooth()` using method = 'gam' and formula = 'y ~ s(x, bs = "cs")'
```

```
ggsave("../output/figs/matching_analysis_bio/additional_expected_island_size_detail_(2,15,25,50).png", height = 3, width = 3)
```

```
## `geom_smooth()` using method = 'gam' and formula = 'y ~ s(x, bs = "cs")'
```

```
ggsave("../output/figs/matching_analysis_bio/additional_expected_island_size_detail_(2,15,25,50).pdf", height = 3, width = 3)
```

```
## `geom_smooth()` using method = 'gam' and formula = 'y ~ s(x, bs = "cs")'
```

## Variable Substitution Frequency Models

```
matching_analysis_simulated_detailed <- read.table("../output/data tables/matching_analysis_simulated_detailed (2,15,25,50)", header=F, sep="&", dec = ".")
colnames(matching_analysis_simulated_detailed) <- c("protocol", "order", "setting", "strobe_fraction", "matches", "sequence_coverage", "match_coverage", "gaps", "mutation_rate", "subs_freq")
matching_analysis_simulated_detailed <- matching_analysis_simulated_detailed %>% mutate(protocol = gsub("mixedrandstrobes", "mixedstrobes", protocol))
matching_analysis_simulated_detailed$protocol <- factor(matching_analysis_simulated_detailed$protocol, levels = c("kmers", "minstrobes", "hybridstrobes", "randstrobes", "mixedstrobes", "altstrobes", "spaced_kmers_dense", "spaced_kmers_sparse", "multistrobes"))
matching_analysis_simulated_detailed <- matching_analysis_simulated_detailed %>% filter(protocol != "mixedstrobes" | strobe_fraction == 0.8)


p <- ggplot(matching_analysis_simulated_detailed %>% filter(order == 2) )+
  geom_point(aes(x = 100*subs_freq, y = matches, col = protocol, shape = protocol))+
  geom_line(aes(x = 100*subs_freq, y = matches, col = protocol))+
  theme(axis.text.x = element_text(angle = 90, vjust = 0.5, hjust=1))+
  theme(legend.position = "none")+
  scale_shape_manual(values = c(16,22,23,24,24,25,17,18,19))+
  scale_color_manual(values = c("#F8766D", "#A3A500", "#00BF7D", "#00B0F6", "blue", "#E76BF3", "gray40", "grey60", "black"))+
  labs(x = "Substitution Frequency [%]",
       y = "Matches [%]")+
  facet_wrap(~mutation_rate, scales = "free")
p +
  scale_x_continuous(sec.axis = sec_axis(~ . , name = "Mutation Rate", breaks = NULL, labels = NULL))
```

```
ggsave("../output/figs/matching_analysis_sim/fig_5_matches_order2_subs_(2,15,25,50).png", height = 2.5, width = 7)
ggsave("../output/figs/matching_analysis_sim/fig_5_matches_order2_subs_(2,15,25,50).pdf", height = 2.5, width = 7)

p <- ggplot(matching_analysis_simulated_detailed %>% filter(order == 2) )+
  geom_point(aes(x = 100*subs_freq, y = sequence_coverage, col = protocol, shape = protocol))+
  geom_line(aes(x = 100*subs_freq, y = sequence_coverage, col = protocol))+
  theme(axis.text.x = element_text(angle = 90, vjust = 0.5, hjust=1))+
  theme(legend.position = "none")+
  scale_shape_manual(values = c(16,22,23,24,24,25,17,18,19))+
  scale_color_manual(values = c("#F8766D", "#A3A500", "#00BF7D", "#00B0F6", "blue", "#E76BF3", "gray40", "grey60", "black"))+
  labs(x = "Substitution Frequency [%]",
       y = "Sequence Coverage [%]")+
  facet_wrap(~mutation_rate, scales = "free")
p +
  scale_x_continuous(sec.axis = sec_axis(~ . , name = "Mutation Rate", breaks = NULL, labels = NULL))
```

```
ggsave("../output/figs/matching_analysis_sim/fig_5_sequence_coverage_order2_subs_(2,15,25,50).png", height = 2.5, width = 7)
ggsave("../output/figs/matching_analysis_sim/fig_5_sequence_coverage_order2_subs_(2,15,25,50).pdf", height = 2.5, width = 7)

p <- ggplot(matching_analysis_simulated_detailed %>% filter(order == 2) )+
  geom_point(aes(x = 100*subs_freq, y = match_coverage, col = protocol, shape = protocol))+
  geom_line(aes(x = 100*subs_freq, y = match_coverage, col = protocol))+
  theme(axis.text.x = element_text(angle = 90, vjust = 0.5, hjust=1))+
  theme(legend.position = "none")+
  scale_shape_manual(values = c(16,22,23,24,24,25,17,18,19))+
  scale_color_manual(values = c("#F8766D", "#A3A500", "#00BF7D", "#00B0F6", "blue", "#E76BF3", "gray40", "grey60", "black"))+
  labs(x = "Substitution Frequency [%]",
       y = "Match Coverage [%]")+
  facet_wrap(~mutation_rate, scales = "free")
p +
  scale_x_continuous(sec.axis = sec_axis(~ . , name = "Mutation Rate", breaks = NULL, labels = NULL))
```

```
ggsave("../output/figs/matching_analysis_sim/fig_5_match_coverage_order2_subs_(2,15,25,50).png", height = 2.5, width = 7)
ggsave("../output/figs/matching_analysis_sim/fig_5_match_coverage_order2_subs_(2,15,25,50).pdf", height = 2.5, width = 7)

p <- ggplot(matching_analysis_simulated_detailed %>% filter(order == 2) )+
  geom_point(aes(x = 100*subs_freq, y = gaps, col = protocol, shape = protocol))+
  geom_line(aes(x = 100*subs_freq, y = gaps, col = protocol))+
  theme(axis.text.x = element_text(angle = 90, vjust = 0.5, hjust=1))+
  theme(legend.position = "none")+
  scale_shape_manual(values = c(16,22,23,24,24,25,17,18,19))+
  scale_color_manual(values = c("#F8766D", "#A3A500", "#00BF7D", "#00B0F6", "blue", "#E76BF3", "gray40", "grey60", "black"))+
  labs(x = "Substitution Frequency [%]",
       y = "Expected Island Size [bp]")+
  facet_wrap(~mutation_rate, scales = "free")
p +
  scale_x_continuous(sec.axis = sec_axis(~ . , name = "Mutation Rate", breaks = NULL, labels = NULL))
```

```
ggsave("../output/figs/matching_analysis_sim/fig_5_expected_island_size_order2_subs_(2,15,25,50).pdf", height = 2.5, width = 7)
ggsave("../output/figs/matching_analysis_sim/fig_5_expected_island_size_order2_subs_(2,15,25,50).png", height = 2.5, width = 7)


results_sim_subs_only <- read.table(file = "../output/data tables/matching_analysis_simulated_subs_only (2,15,25,50)", sep = "&", header = F)
colnames(results_sim_subs_only) <- c("protocol", "order", "setting", "strobe_fraction", "matches", "sequence_coverage", "match_coverage", "gaps", "mutation_rate")
results_sim_subs_only <- results_sim_subs_only %>% mutate(protocol = gsub("mixed", "", protocol))
results_sim_subs_only <- results_sim_subs_only %>% group_by(protocol, order, strobe_fraction, mutation_rate) %>% 
  summarise(matches = mean(matches), sequence_coverage = mean(sequence_coverage), match_coverage = mean(match_coverage), gaps = mean(gaps))
```

```
## `summarise()` has grouped output by 'protocol', 'order', 'strobe_fraction'. You
## can override using the `.groups` argument.
```

```
results_sim_subs_only$protocol <- factor(results_sim_subs_only$protocol, levels = c("kmers", "minstrobes", "hybridstrobes", "randstrobes", "altstrobes", "spaced_kmers_dense", "spaced_kmers_sparse"))
results_sim_subs_only <- results_sim_subs_only %>% filter(protocol != "altstrobes" | strobe_fraction != 0.5)


p <- ggplot(results_sim_subs_only %>% filter(order == 2) )+
  geom_point(aes(x = 100*strobe_fraction, y = matches, col = protocol, shape = protocol))+
  geom_line(aes(x = 100*strobe_fraction, y = matches, col = protocol))+
  theme(axis.text.x = element_text(angle = 90, vjust = 0.5, hjust=1))+
  theme(legend.position = "none")+
  scale_shape_manual(values = c(16,22,23,24,25,17,18))+
  scale_color_manual(values = c("#F8766D", "#A3A500", "#00BF7D", "#00B0F6", "#E76BF3", "gray20", "grey60"))+
  labs(x = "Strobe Fraction [%]",
       y = "Matches [%]")+
  facet_wrap(~mutation_rate, scales = "free")
p +
  scale_x_continuous(sec.axis = sec_axis(~ . , name = "Mutation Rate", breaks = NULL, labels = NULL))
```

```
ggsave("../output/figs/matching_analysis_sim/additional_matches_order2_subs_only_(2,15,25,50).png", height = 3, width = 7)
ggsave("../output/figs/matching_analysis_sim/additional_matches_order2_subs_only_(2,15,25,50).pdf", height = 3, width = 7)

p <- ggplot(results_sim_subs_only %>% filter(order == 2) )+
  geom_point(aes(x = 100*strobe_fraction, y = sequence_coverage, col = protocol, shape = protocol))+
  geom_line(aes(x = 100*strobe_fraction, y = sequence_coverage, col = protocol))+
  theme(axis.text.x = element_text(angle = 90, vjust = 0.5, hjust=1))+
  theme(legend.position = "none")+
  scale_shape_manual(values = c(16,22,23,24,25,17,18))+
  scale_color_manual(values = c("#F8766D", "#A3A500", "#00BF7D", "#00B0F6", "#E76BF3", "gray20", "grey60"))+
  labs(x = "Strobe Fraction [%]",
       y = "Sequence Coverage [%]")+
  facet_wrap(~mutation_rate, scales = "free")
p +
  scale_x_continuous(sec.axis = sec_axis(~ . , name = "Mutation Rate", breaks = NULL, labels = NULL))
```

```
ggsave("../output/figs/matching_analysis_sim/additional_sequence_coverage_order2_subs_only_(2,15,25,50).png", height = 3, width = 7)
ggsave("../output/figs/matching_analysis_sim/additional_sequence_coverage_order2_subs_only_(2,15,25,50).pdf", height = 3, width = 7)

p <- ggplot(results_sim_subs_only %>% filter(order == 2) )+
  geom_point(aes(x = 100*strobe_fraction, y = match_coverage, col = protocol, shape = protocol))+
  geom_line(aes(x = 100*strobe_fraction, y = match_coverage, col = protocol))+
  theme(axis.text.x = element_text(angle = 90, vjust = 0.5, hjust=1))+
  theme(legend.position = "none")+
  scale_shape_manual(values = c(16,22,23,24,25,17,18))+
  scale_color_manual(values = c("#F8766D", "#A3A500", "#00BF7D", "#00B0F6", "#E76BF3", "gray20", "grey60"))+
  labs(x = "Strobe Fraction [%]",
       y = "Match Coverage [%]")+
  facet_wrap(~mutation_rate, scales = "free")
p +
  scale_x_continuous(sec.axis = sec_axis(~ . , name = "Mutation Rate", breaks = NULL, labels = NULL))
```

```
ggsave("../output/figs/matching_analysis_sim/additional_match_coverage_order2_subs_only_(2,15,25,50).png", height = 3, width = 7)
ggsave("../output/figs/matching_analysis_sim/additional_match_coverage_order2_subs_only_(2,15,25,50).pdf", height = 3, width = 7)

p <- ggplot(results_sim_subs_only %>% filter(order == 2) )+
  geom_point(aes(x = 100*strobe_fraction, y = gaps, col = protocol, shape = protocol))+
  geom_line(aes(x = 100*strobe_fraction, y = gaps, col = protocol))+
  theme(axis.text.x = element_text(angle = 90, vjust = 0.5, hjust=1))+
  theme(legend.position = "none")+
  scale_shape_manual(values = c(16,22,23,24,25,17,18))+
  scale_color_manual(values = c("#F8766D", "#A3A500", "#00BF7D", "#00B0F6", "#E76BF3", "gray20", "grey60"))+
  labs(x = "Strobe Fraction [%]",
       y = "Expected Island Size [bp]")+
  facet_wrap(~mutation_rate, scales = "free")
p +
  scale_x_continuous(sec.axis = sec_axis(~ . , name = "Mutation Rate", breaks = NULL, labels = NULL))
```

```
ggsave("../output/figs/matching_analysis_sim/additional_expected_island_size_order2_subs_only_(2,15,25,50).pdf", height = 3, width = 7)
ggsave("../output/figs/matching_analysis_sim/additional_expected_island_size_order2_subs_only_(2,15,25,50).png", height = 3, width = 7)
```

## Strobemers Improve ANI Estimation

```
ANI_simulations <- read.table("../output/data tables/ANI_logger", sep="&", header= FALSE)

strobemers_correction_factor <- 0.17
kmers_correction_factor <- 0.075
spaced_kmers_dense_correction_factor <- 0.3
spaced_kmers_sparse_correction_factor <- 0.6

ANI_simulations <- ANI_simulations %>% mutate(V4 = 1-V4) %>% separate_rows(V3, sep=",") %>% mutate(V3 = as.numeric(V3)) %>% filter(V3 > 0) %>% mutate(
  V1 = ifelse(V1 == "mixedrandstrobes ", "mixedstrobes ", V1),
  V5 = ifelse(V1 == "kmers ", V3 + kmers_correction_factor*(1-V3), 0),
  V5 = ifelse(V1 == "spaced_kmers_dense ", V3 + spaced_kmers_dense_correction_factor*(1-V3), V5),
  V5 = ifelse(V1 == "spaced_kmers_sparse ", V3 + spaced_kmers_sparse_correction_factor*(1-V3), V5),
  V5 = ifelse(V1 %in% c("multistrobes ", "altstrobes ", "mixedstrobes ", "randstrobes ", "minstrobes ", "hybridstrobes "), V3 + strobemers_correction_factor*(1-V3), V5)
)

normalize_kmers <- nrow(ANI_simulations %>% filter(V1 == "kmers "))

ANI_correlation <- ANI_simulations %>% mutate(diff2 = (V5-V4)^2) %>% group_by(V1) %>% summarise(tss = sum(diff2)*normalize_kmers/n(), r = cor(V4, V5, method = "pearson")) %>% mutate(R2 = round(r*r, 2), tss = round(tss, 2)) 
results_ANI_simulations <- ANI_simulations %>% 
  left_join(ANI_correlation, by = c("V1"))

results_ANI_simulations$V1 <- factor(results_ANI_simulations$V1, levels = c("kmers ", "spaced_kmers_dense ", "spaced_kmers_sparse ", "minstrobes ", "hybridstrobes ", "randstrobes ", "mixedstrobes ", "altstrobes ", "multistrobes "))

ggplot(results_ANI_simulations)+
  geom_boxplot(aes(x=V4, y=V5, group = V4, col = V1))+
  geom_abline(slope = 1)+
  geom_text(aes(x= 0.95, y=0.87, hjust = 0, label=paste("R² = ", R2, sep = "")))+
  geom_text(aes(x= 0.95, y=0.82, hjust = 0, label=paste("TSS = ", tss, sep = "")))+
  theme(axis.text.x = element_text(angle = 45, vjust = 0.5, hjust=0.5),
        legend.position = "none")+
  xlab("1 - mutation rate")+ylab("ANI Estimation")+
  scale_color_manual(values = c("#F8766D", "gray40", "grey60", "#A3A500", "#00BF7D", "#00B0F6", "blue", "#E76BF3", "black"))+
  facet_wrap(~ V1)
```

```
ggsave(filename = "../output/figs/ANI/fig_6_ANI_Simulated.pdf", width = 7.5, height = 4.5)
ggsave(filename = "../output/figs/ANI/fig_6_ANI_Simulated.png", width = 7.5, height = 4.5)
```

```
strobemers_correction_factor <- 0.17
kmers_correction_factor <- 0.075
#spaced_kmers_dense_correction_factor <- 0.3
#spaced_kmers_sparse_correction_factor <- 0.6

ANI_biological_kmers <- read.table("../output/data tables/matching_analysis_bio_collinear/kmers.txt", sep="&", header= FALSE) %>% mutate(method = "kmers")
ANI_biological_hybridstrobes <- read.table("../output/data tables/matching_analysis_bio_collinear/hybridstrobe.txt", sep="&", header= FALSE) %>% mutate(method = "hybridstrobes")
ANI_biological_minstrobes <- read.table("../output/data tables/matching_analysis_bio_collinear/minstrobe.txt", sep="&", header= FALSE) %>% mutate(method = "minstrobes")
ANI_biological_mixedstrobes <- read.table("../output/data tables/matching_analysis_bio_collinear/mixedrandstrobe80.txt", sep="&", header= FALSE) %>% mutate(method = "mixedstrobes")
ANI_biological_multistrobes <- read.table("../output/data tables/matching_analysis_bio_collinear/multistrobe.txt", sep="&", header= FALSE) %>% mutate(method = "multistrobes")
ANI_biological_randstrobes <- read.table("../output/data tables/matching_analysis_bio_collinear/randstrobe.txt", sep="&", header= FALSE) %>% mutate(method = "randstrobes")
ANI_biological_altstrobes <- read.table("../output/data tables/matching_analysis_bio_collinear/altstrobe.txt", sep="&", header= FALSE) %>% mutate(method = "altstrobes")

ANI_biological <- rbind(ANI_biological_kmers, ANI_biological_hybridstrobes, ANI_biological_minstrobes, ANI_biological_mixedstrobes, ANI_biological_multistrobes, ANI_biological_randstrobes, ANI_biological_altstrobes) %>% 
  filter(V5/V6 > 0) %>% rowwise %>% 
  mutate(ANI = 1 + 1/30 * log2((2*V5/V6)/(1+V5/V6)),
         ANI = ifelse(method == "kmers", ANI + kmers_correction_factor*(1-ANI), ANI),
         ANI = ifelse(method %in% c("multistrobes", "altstrobes", "mixedstrobes", "randstrobes", "minstrobes", "hybridstrobes"), ANI + strobemers_correction_factor*(1-ANI), ANI),
         TSS = (ANI-0.886)^2)

normalize_kmers <- nrow(ANI_biological %>% filter(method == "kmers"))

results_ANI_biological <- left_join(
  ANI_biological, 
  ANI_biological %>% group_by(method) %>% summarise(meanANI = round(mean(ANI), 3), totalTSS = round(sum(TSS)*normalize_kmers/n(), 1)),
  by = c("method"))
results_ANI_biological$method <- factor(results_ANI_biological$method, levels = c("kmers", "minstrobes", "hybridstrobes", "randstrobes", "mixedstrobes", "altstrobes",  "multistrobes"))

ggplot(results_ANI_biological)+
  geom_violin(aes(x = method, y = ANI, fill=method))+
  geom_text(aes(x = method, y = 1.02, label=paste("ANI=", meanANI, sep = "")))+
  geom_text(aes(x = method, y = 1.00, label=paste("TSS=", totalTSS, sep = "")))+
  geom_hline(yintercept = 0.886, linetype = "dashed")+
  theme(axis.text.x = element_text(angle = 45, vjust = 0.5, hjust=0.5, size = 14),
        legend.position = "none")+
  scale_fill_manual(values = c("#F8766D", "#A3A500", "#00BF7D", "#00B0F6", "blue", "#E76BF3", "gray20"))+
  xlab("")+ylab("ANI Estimation")+
  ylim(0.65, 1.02)+
  ggtitle("Reads-to-Genome Distance: 88.6% (GGDC 3.0)") +
  theme(plot.title = element_text(hjust = 0.9, vjust = -70))
```

```
ggsave(filename = "../output/figs/ANI/fig_7_ANI_Biological_ONTreads.pdf", width = 7.5, height = 4.5)
ggsave(filename = "../output/figs/ANI/fig_7_ANI_Biological_ONTreads.png", width = 7.5, height = 4.5)
```

## Time and Memory to Construct Altstrobes, Mixedstrobes and Multistrobes

```
results_benchmarking_strobemap <- read.table("../output/data tables/benchmark_strobemap_indexing", header=T, sep="&", dec = ".", check.names = FALSE)
plotted_variables <- c("Total indexing time [s]", "Total time generating mers [s]", "Total size of hash table index [Mb]")

results_benchmarking_strobemap$seeding <- factor(results_benchmarking_strobemap$seeding, levels = c("k-mers", "randstrobes", "mixedstrobes", "altstrobes", "multistrobes"))
results_benchmarking_strobemap_melted <- reshape2::melt(results_benchmarking_strobemap, id = c("setting", "genome", "seeding"))
results_benchmarking_strobemap_melted$variable <- factor(results_benchmarking_strobemap_melted$variable, 
                                                         levels=c(
                                                          "Total indexing time [s]",
                                                          "Total time generating mers [s]",
                                                          "Total time generating flat index vector [s]",
                                                          "Total time generating hash table [s]",
                                                          "Maximum resident set size [Mb]",
                                                          "Total size of flat mers-vector [Mb]",
                                                          "Total size of hash table index [Mb]"
                                                          ))
results_benchmarking_strobemap_melted <- results_benchmarking_strobemap_melted %>% filter(variable %in% plotted_variables)

ggplot(results_benchmarking_strobemap_melted %>% filter(genome == "Human Chr.21"))+
  geom_col(aes(x = seeding, y = value/10, fill=seeding))+
  scale_fill_manual(values = c("#F8766D", "#00B0F6", "blue", "#E76BF3", "black"))+
  theme(axis.text.x = element_text(angle = 90, vjust = 0.5, hjust=1),
        axis.title.x=element_blank(),
        axis.title.y=element_blank(),
        legend.position = "none")+
  facet_grid(variable~genome, labeller = label_wrap_gen(width=14), scales="free_y")
```

```
ggsave("../output/figs/benchmarking_strobemap/fig_8_benchmarking_strobemap_human.pdf", width = 3.55, height = 4)
ggsave("../output/figs/benchmarking_strobemap/fig_8_benchmarking_strobemap_human.png", width = 3.55, height = 4)

ggplot(results_benchmarking_strobemap_melted %>% filter(genome == "E.coli"))+
  geom_col(aes(x = seeding, y = value/10, fill=seeding))+
  scale_fill_manual(values = c("#F8766D", "#00B0F6", "blue", "#E76BF3", "black"))+
  theme(axis.text.x = element_text(angle = 90, vjust = 0.5, hjust=1),
        axis.title.x=element_blank(),
        axis.title.y=element_blank(),
        legend.position = "none")+
  facet_grid(variable~genome, labeller = label_wrap_gen(width=14), scales="free_y")
```

```
ggsave("../output/figs/benchmarking_strobemap/fig_8_benchmarking_strobemap_ecoli.pdf", width = 3.5, height = 4)
ggsave("../output/figs/benchmarking_strobemap/fig_8_benchmarking_strobemap_ecoli.png", width = 3.5, height = 4)
```

## Sequence Matching and Uniquenees Results for Altstrobes

```
altstrobes_uniqueness <- read.table("../output/data tables/altstrobes10000_uniqueness", sep = ";", header = F) %>% 
  mutate(V1 = ifelse(V1 == "altstrobes2", "altstrobes", V1),
         V1 = ifelse(V1 == "randstrobes2", "randstrobes", V1)) %>% dplyr::rename("seeding" = "V1")
altstrobes_uniqueness$V2 <- factor(altstrobes_uniqueness$V2, levels = altstrobes_uniqueness$V2)

ggplot(altstrobes_uniqueness, aes(x=V2, y=V3, group=1))+
  geom_point(aes(col = seeding), stat='summary', fun=sum, size = 2.5)+
  stat_summary(aes(col = seeding), fun=sum, geom="line")+
  theme(axis.text.x = element_text(angle = 90, vjust = 0.5, hjust=1),
        legend.position = "bottom",
        legend.title = element_blank())+
  scale_color_manual(values = c("#E76BF3", "#F8766D", "#00B0F6"))+
  xlab("k")+
  ylab("% unique")+
  ylim(0,100)
```

```
ggsave("../output/figs/uniqueness_esize/suppl_fig_S4_altstrobe_uniqueness.png", width = 4.5, height = 3)
ggsave("../output/figs/uniqueness_esize/suppl_fig_S4_altstrobe_uniqueness.pdf", width = 4.5, height = 3)
```

```
altstrobes_raw_data <- read.table(file = "../output/data tables/altstrobes", sep = "&", header = F)
results_sim_alt <- altstrobes_raw_data %>% group_by(V1, V2, V7) %>% summarise(m = round(mean(V3),2), sc = round(mean(V4),2), mc = round(mean(V5),2), esize = round(mean(V6),2))
```

```
## `summarise()` has grouped output by 'V1', 'V2'. You can override using the
## `.groups` argument.
```

```
colnames(results_sim_alt) <- c("protocol", "strobe_lengths", "mutation_rate", "matches", "sequence_coverage", "match_coverage", "gaps")
results_sim_alt$strobe_lengths <- factor(results_sim_alt$strobe_lengths, levels = c(" (1/29) ", " (2/28) ", " (3/27) ", " (4/26) ", " (5/25) ", " (6/24) ", " (7/23) ", " (8/22) ", " (9/21) ", " (10/20) ", " (11/19) ", " (12/18) ", " (13/17) ", " (14/16) ", " (15/15) "))
results_sim_alt <- results_sim_alt %>% mutate(protocol = ifelse(strobe_lengths == " (15/15) ", "randstrobes", protocol))

p <- ggplot(results_sim_alt, aes(x = strobe_lengths, y = matches, group=1))+
  geom_point(aes(col = protocol))+
  geom_line()+
  stat_summary(geom = "line", col = "#E76BF3")+
  scale_color_manual(values = c("#E76BF3", "#00B0F6"))+
  theme(axis.text.x = element_text(angle = 90, vjust = 0.5, hjust=1))+
  theme(legend.position = "none")+
  labs(x = "Strobe Length",
       y = "Matches [%]")+
  facet_wrap(~mutation_rate, scales = "free")

ggsave("../output/figs/matching_analysis_sim/suppl_fig_S5_matches_altstrobes.png", height = 2.5, width = 7)
```

```
## No summary function supplied, defaulting to `mean_se()`
## No summary function supplied, defaulting to `mean_se()`
## No summary function supplied, defaulting to `mean_se()`
```

```
ggsave("../output/figs/matching_analysis_sim/suppl_fig_S5_matches_altstrobes.pdf", height = 2.5, width = 7)
```

```
## No summary function supplied, defaulting to `mean_se()`
## No summary function supplied, defaulting to `mean_se()`
## No summary function supplied, defaulting to `mean_se()`
```

```
p <- ggplot(results_sim_alt, aes(x = strobe_lengths, y = sequence_coverage, group=1))+
  geom_point(aes(col = protocol))+
  geom_line()+
  stat_summary(geom = "line", col = "#E76BF3")+
  scale_color_manual(values = c("#E76BF3", "#00B0F6"))+
  theme(axis.text.x = element_text(angle = 90, vjust = 0.5, hjust=1))+
  theme(legend.position = "none")+
  labs(x = "Strobe Length",
       y = "Sequence Coverage [%]")+
  facet_wrap(~mutation_rate, scales = "free")

ggsave("../output/figs/matching_analysis_sim/suppl_fig_S5_sequence_coverage_altstrobes.png", height = 2.5, width = 7)
```

```
## No summary function supplied, defaulting to `mean_se()`
## No summary function supplied, defaulting to `mean_se()`
## No summary function supplied, defaulting to `mean_se()`
```

```
ggsave("../output/figs/matching_analysis_sim/suppl_fig_S5_sequence_coverage_altstrobes.pdf", height = 2.5, width = 7)
```

```
## No summary function supplied, defaulting to `mean_se()`
## No summary function supplied, defaulting to `mean_se()`
## No summary function supplied, defaulting to `mean_se()`
```

```
p <- ggplot(results_sim_alt, aes(x = strobe_lengths, y = match_coverage, group=1))+
  geom_point(aes(col = protocol))+
  geom_line()+
  stat_summary(geom = "line", col = "#E76BF3")+
  scale_color_manual(values = c("#E76BF3", "#00B0F6"))+
  theme(axis.text.x = element_text(angle = 90, vjust = 0.5, hjust=1))+
  theme(legend.position = "none")+
  labs(x = "Strobe Length",
       y = "Match Coverage [%]")+
  facet_wrap(~mutation_rate, scales = "free")

ggsave("../output/figs/matching_analysis_sim/suppl_fig_S5_match_coverage_altstrobes.png", height = 2.5, width = 7)
```

```
## No summary function supplied, defaulting to `mean_se()`
## No summary function supplied, defaulting to `mean_se()`
## No summary function supplied, defaulting to `mean_se()`
```

```
ggsave("../output/figs/matching_analysis_sim/suppl_fig_S5_match_coverage_altstrobes.pdf", height = 2.5, width = 7)
```

```
## No summary function supplied, defaulting to `mean_se()`
## No summary function supplied, defaulting to `mean_se()`
## No summary function supplied, defaulting to `mean_se()`
```

```
p <- ggplot(results_sim_alt, aes(x = strobe_lengths, y = gaps, group=1))+
  geom_point(aes(col = protocol))+
  geom_line()+
  stat_summary(geom = "line", col = "#E76BF3")+
  scale_color_manual(values = c("#E76BF3", "#00B0F6"))+
  theme(axis.text.x = element_text(angle = 90, vjust = 0.5, hjust=1))+
  theme(legend.position = "none")+
  labs(x = "Strobe Length",
       y = "Expected Island Size [bp]")+
  facet_wrap(~mutation_rate, scales = "free")

ggsave("../output/figs/matching_analysis_sim/suppl_fig_S5_expected_island_size_altstrobes.png", height = 2.5, width = 7)
```

```
## No summary function supplied, defaulting to `mean_se()`
## No summary function supplied, defaulting to `mean_se()`
## No summary function supplied, defaulting to `mean_se()`
```

```
ggsave("../output/figs/matching_analysis_sim/suppl_fig_S5_expected_island_size_altstrobes.pdf", height = 2.5, width = 7)
```

```
## No summary function supplied, defaulting to `mean_se()`
## No summary function supplied, defaulting to `mean_se()`
## No summary function supplied, defaulting to `mean_se()`
```

## Sequence Matching Results for Higher Order Strobemers on Simulated Sequences

```
results_sim <- read.table(file = "../output/data tables/matching_analysis_simulated (2,15,25,50)", sep = "&", header = F)
colnames(results_sim) <- c("protocol", "order", "setting", "strobe_fraction", "matches", "sequence_coverage", "match_coverage", "gaps", "mutation_rate")
results_sim <- results_sim %>% mutate(protocol = gsub("mixed", "", protocol))
results_sim <- results_sim %>% group_by(protocol, order, strobe_fraction, mutation_rate) %>% 
  summarise(matches = mean(matches), sequence_coverage = mean(sequence_coverage), match_coverage = mean(match_coverage), gaps = mean(gaps))
```

```
## `summarise()` has grouped output by 'protocol', 'order', 'strobe_fraction'. You
## can override using the `.groups` argument.
```

```
results_sim$protocol <- factor(results_sim$protocol, levels = c("kmers", "minstrobes", "hybridstrobes", "randstrobes", "altstrobes", "spaced_kmers_dense", "spaced_kmers_sparse", "multistrobes"))
results_sim <- results_sim %>% filter(protocol != "altstrobes" | strobe_fraction != 0.5)

p <- ggplot(results_sim %>% filter(!protocol %in% c("spaced_kmers_dense", "spaced_kmers_sparse")))+
  geom_point(aes(x = 100*strobe_fraction, y = matches, col = protocol, shape = protocol))+
  geom_line(aes(x = 100*strobe_fraction, y = matches, col = protocol))+
  theme(axis.text.x = element_text(angle = 90, vjust = 0.5, hjust=1),
        legend.position = "none")+
  scale_shape_manual(values = c(16,22,23,24,25,21))+
  scale_color_manual(values = c("#F8766D", "#A3A500", "#00BF7D", "#00B0F6", "#E76BF3", "black"))+
  labs(x = "Strobe Fraction [%]",
       y = "Matches [%]")+
  facet_grid(mutation_rate~order, scales = "free")
p +
  scale_x_continuous(sec.axis = sec_axis(~ . , name = "Order", breaks = NULL, labels = NULL)) +
  scale_y_continuous(sec.axis = sec_axis(~ . , name = "Mutation Rate", breaks = NULL, labels = NULL))
```

```
ggsave("../output/figs/matching_analysis_sim/suppl_fig_S6_matches_sim_all_(2,15,25,50).png", height = 4.75, width = 5)
ggsave("../output/figs/matching_analysis_sim/suppl_fig_S6_matches_sim_all_(2,15,25,50).pdf", height = 4.75, width = 5)

p <- ggplot(results_sim %>% filter(!protocol %in% c("spaced_kmers_dense", "spaced_kmers_sparse")) )+
  geom_point(aes(x = 100*strobe_fraction, y = sequence_coverage, col = protocol, shape = protocol))+
  geom_line(aes(x = 100*strobe_fraction, y = sequence_coverage, col = protocol))+
  theme(axis.text.x = element_text(angle = 90, vjust = 0.5, hjust=1),
        legend.position = "none")+
  scale_shape_manual(values = c(16,22,23,24,25,21))+
  scale_color_manual(values = c("#F8766D", "#A3A500", "#00BF7D", "#00B0F6", "#E76BF3", "black"))+
  labs(x = "Strobe Fraction [%]",
       y = "Sequence Coverage [%]")+
  facet_grid(mutation_rate~order, scales = "free")
p +
  scale_x_continuous(sec.axis = sec_axis(~ . , name = "Order", breaks = NULL, labels = NULL)) +
  scale_y_continuous(sec.axis = sec_axis(~ . , name = "Mutation Rate", breaks = NULL, labels = NULL))
```

```
ggsave("../output/figs/matching_analysis_sim/suppl_fig_S6_sequence_coverage_sim_all_(2,15,25,50).png", height = 4.75, width = 5)
ggsave("../output/figs/matching_analysis_sim/suppl_fig_S6_sequence_coverage_sim_all_(2,15,25,50).pdf", height = 4.75, width = 5)

p <- ggplot(results_sim %>% filter(!protocol %in% c("spaced_kmers_dense", "spaced_kmers_sparse")) )+
  geom_point(aes(x = 100*strobe_fraction, y = match_coverage, col = protocol, shape = protocol))+
  geom_line(aes(x = 100*strobe_fraction, y = match_coverage, col = protocol))+
  theme(axis.text.x = element_text(angle = 90, vjust = 0.5, hjust=1),
        legend.position = "none")+
  scale_shape_manual(values = c(16,22,23,24,25,21))+
  scale_color_manual(values = c("#F8766D", "#A3A500", "#00BF7D", "#00B0F6", "#E76BF3", "black"))+
  labs(x = "Strobe Fraction [%]",
       y = "Match Coverage [%]")+
  facet_grid(mutation_rate~order, scales = "free")
p +
  scale_x_continuous(sec.axis = sec_axis(~ . , name = "Order", breaks = NULL, labels = NULL)) +
  scale_y_continuous(sec.axis = sec_axis(~ . , name = "Mutation Rate", breaks = NULL, labels = NULL))
```

```
ggsave("../output/figs/matching_analysis_sim/suppl_fig_S6_match_coverage_sim_all_(2,15,25,50).png", height = 4.75, width = 5)
ggsave("../output/figs/matching_analysis_sim/suppl_fig_S6_match_coverage_sim_all_(2,15,25,50).pdf", height = 4.75, width = 5)

p <- ggplot(results_sim %>% filter(!protocol %in% c("spaced_kmers_dense", "spaced_kmers_sparse")) )+
  geom_point(aes(x = 100*strobe_fraction, y = gaps, col = protocol, shape = protocol))+
  geom_line(aes(x = 100*strobe_fraction, y = gaps, col = protocol))+
  theme(axis.text.x = element_text(angle = 90, vjust = 0.5, hjust=1),
        legend.position = "none")+
  scale_shape_manual(values = c(16,22,23,24,25,21))+
  scale_color_manual(values = c("#F8766D", "#A3A500", "#00BF7D", "#00B0F6", "#E76BF3", "black"))+
  labs(x = "Strobe Fraction [%]",
       y = "Expected Island Size [bp]")+
  facet_grid(mutation_rate~order, scales = "free")
p +
  scale_x_continuous(sec.axis = sec_axis(~ . , name = "Order", breaks = NULL, labels = NULL)) +
  scale_y_continuous(sec.axis = sec_axis(~ . , name = "Mutation Rate", breaks = NULL, labels = NULL))
```

```
ggsave("../output/figs/matching_analysis_sim/suppl_fig_S6_expected_island_size_sim_all_(2,15,25,50).png", height = 4.75, width = 5)
ggsave("../output/figs/matching_analysis_sim/suppl_fig_S6_expected_island_size_sim_all_(2,15,25,50).pdf", height = 4.75, width = 5)


p <- ggplot(results_sim %>% filter(!protocol %in% c("spaced_kmers_dense", "spaced_kmers_sparse")) %>% filter(order == 2) )+
  geom_point(aes(x = 100*strobe_fraction, y = matches, col = protocol, shape = protocol))+
  geom_line(aes(x = 100*strobe_fraction, y = matches, col = protocol))+
  theme(axis.text.x = element_text(angle = 90, vjust = 0.5, hjust=1))+
  theme(legend.position = "none")+
  scale_shape_manual(values = c(16,22,23,24,25,21))+
  scale_color_manual(values = c("#F8766D", "#A3A500", "#00BF7D", "#00B0F6", "#E76BF3", "black"))+
  labs(x = "Strobe Fraction [%]",
       y = "Matches [%]")+
  facet_wrap(~mutation_rate, scales = "free")
p +
  scale_x_continuous(sec.axis = sec_axis(~ . , name = "Mutation Rate", breaks = NULL, labels = NULL))
```

```
ggsave("../output/figs/matching_analysis_sim/additional_matches_order2_(2,15,25,50).png", height = 3, width = 7)
ggsave("../output/figs/matching_analysis_sim/additional_matches_order2_(2,15,25,50).pdf", height = 3, width = 7)

p <- ggplot(results_sim %>% filter(!protocol %in% c("spaced_kmers_dense", "spaced_kmers_sparse")) %>% filter(order == 2) )+
  geom_point(aes(x = 100*strobe_fraction, y = sequence_coverage, col = protocol, shape = protocol))+
  geom_line(aes(x = 100*strobe_fraction, y = sequence_coverage, col = protocol))+
  theme(axis.text.x = element_text(angle = 90, vjust = 0.5, hjust=1))+
  theme(legend.position = "none")+
  scale_shape_manual(values = c(16,22,23,24,25,21))+
  scale_color_manual(values = c("#F8766D", "#A3A500", "#00BF7D", "#00B0F6", "#E76BF3", "black"))+
  labs(x = "Strobe Fraction [%]",
       y = "Sequence Coverage [%]")+
  facet_wrap(~mutation_rate, scales = "free")
p +
  scale_x_continuous(sec.axis = sec_axis(~ . , name = "Mutation Rate", breaks = NULL, labels = NULL))
```

```
ggsave("../output/figs/matching_analysis_sim/additional_sequence_coverage_order2_(2,15,25,50).png", height = 3, width = 7)
ggsave("../output/figs/matching_analysis_sim/additional_sequence_coverage_order2_(2,15,25,50).pdf", height = 3, width = 7)

p <- ggplot(results_sim %>% filter(!protocol %in% c("spaced_kmers_dense", "spaced_kmers_sparse")) %>% filter(order == 2) )+
  geom_point(aes(x = 100*strobe_fraction, y = match_coverage, col = protocol, shape = protocol))+
  geom_line(aes(x = 100*strobe_fraction, y = match_coverage, col = protocol))+
  theme(axis.text.x = element_text(angle = 90, vjust = 0.5, hjust=1))+
  theme(legend.position = "none")+
  scale_shape_manual(values = c(16,22,23,24,25,21))+
  scale_color_manual(values = c("#F8766D", "#A3A500", "#00BF7D", "#00B0F6", "#E76BF3", "black"))+
  labs(x = "Strobe Fraction [%]",
       y = "Match Coverage [%]")+
  facet_wrap(~mutation_rate, scales = "free")
p +
  scale_x_continuous(sec.axis = sec_axis(~ . , name = "Mutation Rate", breaks = NULL, labels = NULL))
```

```
ggsave("../output/figs/matching_analysis_sim/additional_match_coverage_order2_(2,15,25,50).png", height = 3, width = 7)
ggsave("../output/figs/matching_analysis_sim/additional_match_coverage_order2_(2,15,25,50).pdf", height = 3, width = 7)

p <- ggplot(results_sim %>% filter(!protocol %in% c("spaced_kmers_dense", "spaced_kmers_sparse")) %>% filter(order == 2) )+
  geom_point(aes(x = 100*strobe_fraction, y = gaps, col = protocol, shape = protocol))+
  geom_line(aes(x = 100*strobe_fraction, y = gaps, col = protocol))+
  theme(axis.text.x = element_text(angle = 90, vjust = 0.5, hjust=1))+
  theme(legend.position = "none")+
  scale_shape_manual(values = c(16,22,23,24,25,21))+
  scale_color_manual(values = c("#F8766D", "#A3A500", "#00BF7D", "#00B0F6", "#E76BF3", "black"))+
  labs(x = "Strobe Fraction [%]",
       y = "Expected Island Size [bp]")+
  facet_wrap(~mutation_rate, scales = "free")
p +
  scale_x_continuous(sec.axis = sec_axis(~ . , name = "Mutation Rate", breaks = NULL, labels = NULL))
```

```
ggsave("../output/figs/matching_analysis_sim/additional_expected_island_size_order2_(2,15,25,50).pdf", height = 3, width = 7)
ggsave("../output/figs/matching_analysis_sim/additional_expected_island_size_order2_(2,15,25,50).png", height = 3, width = 7)


results_sim <- read.table(file = "../output/data tables/matching_analysis_simulated_(2,15,16,50)", sep = "&", header = F)
colnames(results_sim) <- c("protocol", "order", "setting", "strobe_fraction", "matches", "sequence_coverage", "match_coverage", "gaps", "mutation_rate")
results_sim <- results_sim %>% mutate(protocol = gsub("mixed", "", protocol))
results_sim$protocol <- factor(results_sim$protocol, levels = c("kmers", "minstrobes", "hybridstrobes", "randstrobes", "altstrobes", "spaced_kmers_dense", "spaced_kmers_sparse"))
results_sim <- results_sim %>% filter(protocol != "altstrobes" | strobe_fraction != 0.5)

p <- ggplot(results_sim %>% filter(!protocol %in% c("spaced_kmers_dense", "spaced_kmers_sparse")) %>% filter(order == 2) )+
  geom_point(aes(x = 100*strobe_fraction, y = matches, col = protocol, shape = protocol))+
  geom_line(aes(x = 100*strobe_fraction, y = matches, col = protocol))+
  theme(axis.text.x = element_text(angle = 90, vjust = 0.5, hjust=1))+
  theme(legend.position = "none")+
  scale_shape_manual(values = c(16,22,23,24,25))+
  labs(x = "Strobe Fraction [%]",
       y = "Matches [%]")+
  facet_wrap(~mutation_rate, scales = "free")
p +
  scale_x_continuous(sec.axis = sec_axis(~ . , name = "Mutation Rate", breaks = NULL, labels = NULL))
```

```
ggsave("../output/figs/matching_analysis_sim/additional_matches_order2_(2,15,16,50).png", height = 3, width = 7)
ggsave("../output/figs/matching_analysis_sim/additional_matches_order2_(2,15,16,50).pdf", height = 3, width = 7)

p <- ggplot(results_sim %>% filter(!protocol %in% c("spaced_kmers_dense", "spaced_kmers_sparse")) %>% filter(order == 2) )+
  geom_point(aes(x = 100*strobe_fraction, y = sequence_coverage, col = protocol, shape = protocol))+
  geom_line(aes(x = 100*strobe_fraction, y = sequence_coverage, col = protocol))+
  theme(axis.text.x = element_text(angle = 90, vjust = 0.5, hjust=1))+
  theme(legend.position = "none")+
  scale_shape_manual(values = c(16,22,23,24,25))+
  labs(x = "Strobe Fraction [%]",
       y = "Sequence Coverage [%]")+
  facet_wrap(~mutation_rate, scales = "free")
p +
  scale_x_continuous(sec.axis = sec_axis(~ . , name = "Mutation Rate", breaks = NULL, labels = NULL))
```

```
ggsave("../output/figs/matching_analysis_sim/additional_sequence_coverage_order2_(2,15,16,50).png", height = 3, width = 7)
ggsave("../output/figs/matching_analysis_sim/additional_sequence_coverage_order2_(2,15,16,50).pdf", height = 3, width = 7)

p <- ggplot(results_sim %>% filter(!protocol %in% c("spaced_kmers_dense", "spaced_kmers_sparse")) %>% filter(order == 2) )+
  geom_point(aes(x = 100*strobe_fraction, y = match_coverage, col = protocol, shape = protocol))+
  geom_line(aes(x = 100*strobe_fraction, y = match_coverage, col = protocol))+
  theme(axis.text.x = element_text(angle = 90, vjust = 0.5, hjust=1))+
  theme(legend.position = "none")+
  scale_shape_manual(values = c(16,22,23,24,25))+
  labs(x = "Strobe Fraction [%]",
       y = "Match Coverage [%]")+
  facet_wrap(~mutation_rate, scales = "free")
p +
  scale_x_continuous(sec.axis = sec_axis(~ . , name = "Mutation Rate", breaks = NULL, labels = NULL))
```

```
ggsave("../output/figs/matching_analysis_sim/additional_match_coverage_order2_(2,15,16,50).png", height = 3, width = 7)
ggsave("../output/figs/matching_analysis_sim/additional_match_coverage_order2_(2,15,16,50).pdf", height = 3, width = 7)

p <- ggplot(results_sim %>% filter(!protocol %in% c("spaced_kmers_dense", "spaced_kmers_sparse")) %>% filter(order == 2) )+
  geom_point(aes(x = 100*strobe_fraction, y = gaps, col = protocol, shape = protocol))+
  geom_line(aes(x = 100*strobe_fraction, y = gaps, col = protocol))+
  theme(axis.text.x = element_text(angle = 90, vjust = 0.5, hjust=1))+
  theme(legend.position = "none")+
  scale_shape_manual(values = c(16,22,23,24,25))+
  labs(x = "Strobe Fraction [%]",
       y = "Expected Island Size [bp]")+
  facet_wrap(~mutation_rate, scales = "free")
p +
  scale_x_continuous(sec.axis = sec_axis(~ . , name = "Mutation Rate", breaks = NULL, labels = NULL))
```

```
ggsave("../output/figs/matching_analysis_sim/additional_expected_island_size_order2_(2,15,16,50).pdf", height = 3, width = 7)
ggsave("../output/figs/matching_analysis_sim/additional_expected_island_size_order2_(2,15,16,50).png", height = 3, width = 7)
```

## Minimap2 Benchmarking Results

```
results_minimap <- read.table("../output/data tables/minimap_results", header=T, sep="&", dec = ".", check.names = FALSE)

results_minimap_melted <- reshape2::melt(results_minimap %>% unite("seeding", "seeding":"settings", remove = TRUE, sep = " "), id=c("seeding", "mut_freq"))
results_minimap_melted$seeding <- factor(results_minimap_melted$seeding, levels = c("altstrobes (2,9,18,25,50)", "kmers (15)", "kmers (28)", "mixedstrobes (2,14,25,50,0.8)", "randstrobes (2,14,25,50)", "multistrobes (2,6,22,25,50)"))

ggplot(results_minimap_melted)+
  geom_point(aes(x=seeding, y=value, col=seeding), size = 2)+
  theme(axis.text.x = element_text(angle = 90, vjust = 0.5, hjust=1),
        axis.title.x=element_blank(),
        axis.title.y=element_blank(),
        legend.position = "none")+
  #scale_fill_manual(values = c("#E76BF3", "#F8766D", "#F8766D", "blue", "#00B0F6", "black"))+
  scale_color_manual(values = c("#E76BF3", "#F8766D", "#F8766D", "blue", "#00B0F6", "black"))+
  facet_grid(variable ~ mut_freq, scales = "free")
```

```
ggsave("../output/figs/benchmarking_minimap/additional_minimap.pdf", width = 7, height = 18)
ggsave("../output/figs/benchmarking_minimap/additional_minimap.png", width = 7, height = 18)


#unique(results_minimap_melted$variable)
variable_plotted = c("total time [s]", "indexing time [s]", "aligning time [s]", "peak RSS [Gb]", "mapped correctly [%]")

ggplot(results_minimap_melted %>% filter(variable %in% variable_plotted))+
  geom_point(aes(x=seeding, y=value, col=seeding), size = 2)+
  theme(axis.text.x = element_text(angle = 90, vjust = 0.5, hjust=1),
        axis.title.x=element_blank(),
        axis.title.y=element_blank(),
        legend.position = "none")+
  #scale_fill_manual(values = c("#E76BF3", "#F8766D", "#F8766D", "blue", "#00B0F6", "black"))+
  scale_color_manual(values = c("#E76BF3", "#F8766D", "#F8766D", "blue", "#00B0F6", "black"))+
  facet_grid(variable ~ mut_freq, scales = "free")
```

```
ggsave("../output/figs/benchmarking_minimap/suppl_fig_S9_minimap_1.pdf", width = 5.5, height = 9)
ggsave("../output/figs/benchmarking_minimap/suppl_fig_S9_minimap_1.png", width = 5.5, height = 9)


ggplot(results_minimap_melted %>% filter(!variable %in% variable_plotted))+
  geom_point(aes(x=seeding, y=value, col=seeding), size = 2)+
  theme(axis.text.x = element_text(angle = 90, vjust = 0.5, hjust=1),
        axis.title.x=element_blank(),
        axis.title.y=element_blank(),
        legend.position = "none")+
  #scale_fill_manual(values = c("#E76BF3", "#F8766D", "#F8766D", "blue", "#00B0F6", "black"))+
  scale_color_manual(values = c("#E76BF3", "#F8766D", "#F8766D", "blue", "#00B0F6", "black"))+
  facet_grid(variable ~ mut_freq, scales = "free")
```

```
ggsave("../output/figs/benchmarking_minimap/suppl_fig_S9_minimap_2.pdf", width = 5.5, height = 9)
ggsave("../output/figs/benchmarking_minimap/suppl_fig_S9_minimap_2.png", width = 5.5, height = 9)
```

## Seed Uniqueness

```
results_uniqueness <- read.table(file = "../output/data tables/uniqueness.csv", header = TRUE, sep = ",")
results_uniqueness_mixed <- results_uniqueness %>% filter(!Method %in% c("kmers", "multistrobes2")) %>% separate(Method, c("Method", "Order"), sep="(?<=strobes)") %>% mutate(Order = paste("Order",Order))
results_uniqueness_mixed$Method = factor(results_uniqueness_mixed$Method, levels=c('mixedminstrobes', 'mixedhybridstrobes', 'mixedrandstrobes', 'mixedaltstrobes'))

ggplot(results_uniqueness_mixed)+
  geom_line(aes(x = k, y = unique, group= strobefraction, col = strobefraction))+
  theme(legend.position = "bottom")+
  facet_grid(Order ~ Method)+
  scale_color_viridis(discrete = FALSE, option = "B")+
  ylab("% unique")+
  scale_x_continuous(limits = c(18, 36))
```

```
ggsave("../output/figs/uniqueness_esize/suppl_fig_S10_uniqueness_mixed.png", width = 5.3, height = 5)
ggsave("../output/figs/uniqueness_esize/suppl_fig_S10_uniqueness_mixed.pdf", width = 5.3, height = 5)

results_uniqueness2 <- results_uniqueness %>% filter(Method == "kmers" | strobefraction == 100) %>% mutate(Method = str_replace(Method, "mixed",""))
results_uniqueness2$Method = factor(results_uniqueness2$Method, levels=c('kmers', 'minstrobes2', 'minstrobes3', 'hybridstrobes2', 'hybridstrobes3', 'randstrobes2', 'randstrobes3', 'altstrobes2', 'multistrobes2'))                                                                                                           

ggplot(results_uniqueness2)+
  geom_line(aes(x = k, y = unique, col = Method, linetype = Method))+
  geom_point(aes(x = k, y = unique, col = Method, shape = Method))+
  theme(legend.position = "bottom",
        legend.title = element_blank())+
  guides(linetype = guide_legend(nrow = 3, byrow = TRUE))+
  scale_linetype_manual(values=c("solid", "longdash", "dotted", "longdash", "dotted", "longdash", "dotted", "longdash", "longdash"))+
  scale_color_manual(values=c("#F8766D", "#CD9600", "#7CAE00", "#00BE67", "#00BFC4", "#00A9FF", "#C77CFF", "#FF61CC", "black"))+
  scale_shape_manual(values = c(16,22,22,23,23,24,24,25,19))+
  ylab("% unique")+
  scale_x_continuous(limits = c(18, 36))
```

```
ggsave("../output/figs/uniqueness_esize/suppl_fig_S10_uniqueness_overview.png", width = 4, height = 4.5)
ggsave("../output/figs/uniqueness_esize/suppl_fig_S10_uniqueness_overview.pdf", width = 4, height = 4.5)
```

## RECOMB Presentation Figures

```
ggplot(read.csv("../output/data tables/kmers_recomb.csv", sep = "\t"))+
  geom_line(aes(x = mutation_rate, y=coverage), col="#F8766D", size = 1.5)+
  geom_abline(intercept = 100, slope = -1, linetype = "dashed")+
  ylim(0, 100)+
  xlab("Mutation Rate [%]")+
  ylab("Sequence Coverage [%]")
```

```
ggsave("~/Desktop/matching_analysis_sim/RECOMB_kmerreasoning.png", width = 2.5, height = 2.5)

ggplot(matching_analysis_simulated_detailed %>% filter(order == 2, mutation_rate == 0.05, protocol %in% c("spaced_kmers_dense", "spaced_kmers_sparse")) %>% mutate(mutation_rate = paste("Mutation Rate =", mutation_rate)))+
  geom_line(aes(x = 100*(1-subs_freq), y = sequence_coverage, col = protocol), size = 1.5)+
  theme(axis.text.x = element_text(angle = 90, vjust = 0.5, hjust=1))+
  theme(legend.position = "none")+
  scale_shape_manual(values = c(17,18))+
  scale_color_manual(values = c("gray40", "grey60"))+
  labs(x = "InDel Frequency [%]",
       y = "Sequence Coverage [%]")+
  facet_wrap(~mutation_rate, scales = "free")
```

```
ggsave("../output/figs/matching_analysis_sim/RECOMB_sequence_coverage_order2_subs_(2,15,25,50).png", width = 2.25, height = 2.25)
```

```
results_minimap <- read.table("../output/data tables/minimap_results", header=T, sep="&", dec = ".", check.names = FALSE)

results_minimap_melted <- reshape2::melt(results_minimap %>% unite("seeding", "seeding":"settings", remove = TRUE, sep = " "), id=c("seeding", "mut_freq"))
results_minimap_melted$seeding <- factor(results_minimap_melted$seeding, levels = c("altstrobes (2,9,18,25,50)", "kmers (15)", "kmers (28)", "mixedstrobes (2,14,25,50,0.8)", "randstrobes (2,14,25,50)", "multistrobes (2,6,22,25,50)"))

#unique(results_minimap_melted$variable)
#variable_plotted = c("total time [s]", "indexing time [s]", "aligning time [s]")
variable_plotted = c("distinct minimizers", "singletons [%]", "mapped correctly [%]")

ggplot(results_minimap_melted %>% filter(variable %in% variable_plotted))+
  geom_point(aes(x=seeding, y=value, col=seeding), size = 2)+
  theme(axis.text.x = element_blank(),
        axis.title.x=element_blank(),
        axis.title.y=element_blank(),
        legend.position = "right")+
  #scale_fill_manual(values = c("#E76BF3", "#F8766D", "#F8766D", "blue", "#00B0F6", "black"))+
  scale_color_manual(values = c("#E76BF3", "red", "#F8766D", "blue", "#00B0F6", "black"))+
  facet_grid(variable ~ mut_freq, scales = "free")
```

```
ggsave("../output/figs/benchmarking_minimap/RECOMB_minimap.png", width = 7.8, height = 4.5)
```
